# Decoding MHC loss: Molecular mechanisms and implications for immune resistance in cancer

**DOI:** 10.1002/ctm2.70403

**Published:** 2025-07-17

**Authors:** Pei Lin, Yunfan Lin, Xu Chen, Xinyuan Zhao, Li Cui

**Affiliations:** ^1^ School of Stomatology Stomatological Hospital Southern Medical University Guangzhou Guangdong China; ^2^ School of Dentistry University of California, Los Angeles Los Angeles California USA

**Keywords:** cancer immunotherapy, immune responses, MHC loss, tumour microenvironment

## Abstract

Loss or downregulation of major histocompatibility complex (MHC) molecules represents a key mechanism by which tumours escape immune recognition and acquire resistance to immunotherapeutic interventions. This review focuses on the central regulatory pathways. These includes transcriptional repression, lysosomal degradation, and post‐translational modifications that disrupt MHC stability, trafficking, and surface expression. We highlight how these mechanisms impair antigen presentation and contribute to tumour immune evasion. In addition, we explore emerging therapeutic strategies focused on reactivating MHC expression to enhance tumour immunogenicity and improve the efficacy of immunotherapy. Finally, we discuss the translational potential of these approaches and the remaining challenges, including tumour heterogeneity, immunotoxicity and dynamic regulation within the tumour microenvironment, that must be addressed to optimize MHC‐targeted interventions in cancer immunotherapy.

**Highlights:**

- Tumour cells evade immune surveillance by downregulating MHC expression through transcriptional repression, lysosomal degradation and post‐translational modifications.
- Pharmacological agents interventing epigenetic and metabolic can upregulate MHC expression and improve T cell activation.
- Combination strategies potentiate immunotherapy efficacy by reinvigorating tumour immunogenicity.

## Graphical abstract

1. Tumour cells evade immune surveillance by downregulating MHC expression through transcriptional repression, lysosomal degradation and post‐translational modifications.
2. Pharmacological agents interventing epigenetic and metabolic can upregulate MHC expression and improve T cell activation.
3. Combination strategies potentiate immunotherapy efficacy by reinvigorating tumour immunogenicity.

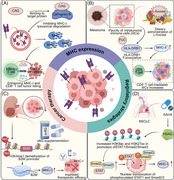

## 1 INTRODUCTION

Cancer continues to be a major contributor to global morbidity and mortality, imposing a significant burden on public health despite ongoing progress in early detection and treatment. Recently, approximately 2 001 140 new cancer cases and 611 720 deaths are projected to occur in the U.S. While mortality has declined—averting over 4 million deaths since 1991—incidence is rising for several major cancers, including colorectal cancer, now representing the leading cause of cancer‐related death in men under 50. The overall 5‐year survival rate for all cancers has improved to 69%, but remains low for pancreatic (13%), liver (22%) and lung cancers (25%). These trends underscore the pressing need for continued cancer research and innovative therapeutic strategies.^1^

Contemporary cancer immunotherapy has rapidly evolved beyond traditional immune checkpoint blockade (ICB) to encompass a broader array of strategies. Progress in ICB has highlighted the importance of context‐specific biomarkers and tumour microenvironmental features in guiding therapeutic decisions. For instance, in gastric cancer, evolving insights into the PD‐1/PD‐L1 axis underscore the potential of combinatorial approaches targeting additional checkpoints and tailoring treatment based on tumour microenvironment profiling.^2^ In addition, circulating biomarkers such as soluble PD‐L1, cytokine profiles, and lymphocyte subsets have demonstrated potential in predicting response to PD‐1/PD‐L1 blockade.^3^ Advances in cell engineering, such as CAR‐T and TCR‐T therapies, have enabled precise targeting of tumour antigens. Strategies incorporating genetic modifications and synthetic biology approaches to address the metabolic constraints within the tumour microenvironment (TME) may pave the way for more effective and durable responses to cell engineering therapies in solid tumours.^4^ Meanwhile, innovations in microenvironmental modulation aim to overcome immunosuppression through reprogramming of stromal and myeloid components. For instance, interrupting the crosstalk between tumour cells and tumour‐associated macrophages (TAMs) mediated by small extracellular vesicles offers a novel avenue to reprogram immunosuppressive macrophages and bolster anti‐tumour immunity.^5^ Together, these approaches complement checkpoint inhibition and offer synergistic potential for durable and personalized therapy. Looking ahead, cancer therapy is moving toward more personalized and precise approaches. Progress in genomics and molecular profiling enable treatments tailored to individual tumour characteristics. Integrating immunotherapy with targeted therapies aim to overcome resistance and improve efficacy. Additionally, innovations in nanomedicine and gene editing hold promise for enhancing drug delivery and targeting. Overall, the future of cancer treatment focuses on maximizing effectiveness while minimizing side effects, with an emphasis on equitable access and addressing tumour heterogeneity.^6^, ^7^

Major histocompatibility complex (MHC) molecules are essential to the immune system, serving a central role in presenting antigens to T cells.^8^, ^9^ These glycoproteins are categorized into two main classes: Class I MHC (MHC‐I) and Class II MHC (MHC‐II).^10^ MHC‐I, expressed on the surface of nearly all nucleated cells, presents endogenous peptides, typically derived from intracellular proteins, to CD8^+^ cytotoxic T cells.^11^ In contrast, MHC‐II is primarily expressed on antigen‐presenting cells (APCs), such as dendritic cells (DCs), macrophages, and B cells, where they display exogenous peptides to CD4^+^ helper T cells.^12^

The ability of MHC to bind and present a diverse array of peptides is governed by their polymorphic nature, which allows for the recognition of a wide variety of pathogens.^13^, ^14^ This diversity is essential for the adaptive immunity because it enables the immune system to identify and combat a broad spectrum of infections and diseases.^15^, ^16^ For instance, SARS‐CoV‐2′s open reading frame 8 (ORF8) protein interacts with MHC‐I, leading to their degradation via autophagy‐mediated lysosomal pathways. This reduces the visibility of infected cells to cytotoxic T lymphocytes (CTLs), undermining antigen presentation. Targeting ORF8 may therefore enhance immune detection of the virus.^17^ Moreover, the interaction between MHC and T cell receptors (TCRs) is crucial for immune surveillance, tolerance, and the orchestration of both innate and adaptive immune responses.^18^, ^19^ Dysregulation of MHC expression or function is implicated in various autoimmune disorders, transplant rejection, and the evasion of immune surveillance by tumours.^20^, ^21^, ^22^, ^23^, ^24^ Thus, MHC molecules are central to both immune defence and immune regulation, with significant implications for disease pathogenesis and therapeutic strategies.

Here, we examine how MHC loss at multiple levels impairs immune therapy and highlight pathways to restore MHC expression. The mechanisms by which MHC loss at the transcriptional regulation level influences immune therapy are first explored. The role of lysosomal and post‐transcriptional modification‐mediated MHC degradation is then analysed, focusing on how these mechanisms influence MHC stability, trafficking, and surface expression, ultimately affecting immune recognition and tumour evasion. The review also investigates how MHC loss contributes to resistance against immune therapies and emphasizes the importance of restoring MHC expression to overcome treatment failure. Finally, current challenges and future strategies for addressing MHC loss in immunotherapy are discussed, with insights into potential solutions to enhance treatment efficacy.

## 2 LOSS OF MHC EXPRESSION AS A CRITICAL FACTOR INFLUENCING CANCER IMMUNE ESCAPE

### 2.1 Functional consequences of MHC downregulation in tumours

MHC loss is a prominent factor contributing to cancer progression and resistance to immunotherapy.^25^, ^26^ A clinical study reported that among 181 melanoma patients, 78 cases (43%) exhibited either complete or predominant (> 50%) loss of MHC‐I expression, which was linked to reduced transcription of HLA‐A, HLA‐B, HLA‐C and B2M, and closely linked to primary resistance to anti‐CTLA‐4 therapy. Conversely, patients with MHC‐II expression in more than 1% of tumour cells (30%) showed correlation with interferon‐γ (IFN‐γ) and IFN‐γ–mediated gene signatures, and demonstrated favourable responses to anti‐PD‐1 treatment.^27^ MHC molecules present antigen to T cells, facilitating immune surveillance. Their downregulation impairs antigen presentation to CD8⁺ and CD4⁺ T cells, promoting immune escape and compromising the effectiveness of T cell‐based immunotherapies, including checkpoint blockade, adoptive T cell therapy and cancer vaccines.^28^, ^29^ For example, MAL2 promotes antigen internalization and reduces MHC‐I surface levels, limiting CD8⁺ T cell recognition in breast cancer. Inhibiting MAL2 enhances CD8⁺ T cell cytotoxicity and suppresses tumour growth, underscoring its relevance to immune evasion and potential therapeutic targeting.^30^ Similarly, GR signalling in pancreatic ductal adenocarcinoma (PDAC) suppresses MHC‐I and upregulates PD‐L1 expression, impairing T cell surveillance. Pharmacological inhibition of GR restores MHC‐I levels and improves responsiveness to ICB.^31^ These findings highlight the clinical significance of understanding and overcoming MHC loss.

### 2.2 Genetic and epigenetic mechanisms driving MHC loss

The loss of MHC expression involves both genetic and epigenetic mechanisms. Mutations in the beta‐2‐microglobulin (B2M) gene or in components of the antigen‐processing machinery (e.g., TAP1/2, ERAP1/2) can disrupt peptide loading and MHC‐I surface expression, contributing to immune evasion.^32^ Epigenetic silencing of MHC genes through promoter hypermethylation or histone deacetylation further impairs their transcription.^33^, ^34^ Additionally, chromatin architecture plays a key role: super enhancers such as DR/DQ‐SE and XL9‐SE at the MHC‐II locus regulate gene accessibility. CRISPR‐mediated deletion of these elements impairs MHC‐II gene expression and chromatin structure, suggesting potential epigenetic targets for restoring MHC expression in tumours.^35^ Tumours may also acquire mutations in transcriptional regulators like interferon regulatory factors (IRFs) or components of the NF‐κB pathway, disrupting IFN‐induced MHC upregulation.^36^ A recent pan‐cancer TCGA‐based analysis comprehensively characterized the genomic and clinical profiles of IRF family genes across 33 tumour types, revealing that alterations such as SNPs, CNVs, and DNA methylation drive IRF dysregulation. These changes were shown to influence tumour progression, immune infiltration and drug sensitivity, highlighting IRFs as critical transcriptional regulators and potential predictors of therapeutic efficacy.^37^ These multifaceted regulatory disruptions collectively impair antigen presentation and enable immune evasion. While epigenetic silencing enables dynamic but reversible suppression of MHC, it operates more slowly than post‐translational modifications like ubiquitination, which offer a rapid, proteasome‐directed switch‐off of MHC surface expression. Understanding these layered mechanisms highlights distinct therapeutic windows for restoring antigen presentation.

### 2.3 Microenvironmental factors influencing MHC expression

**FIGURE 1:**
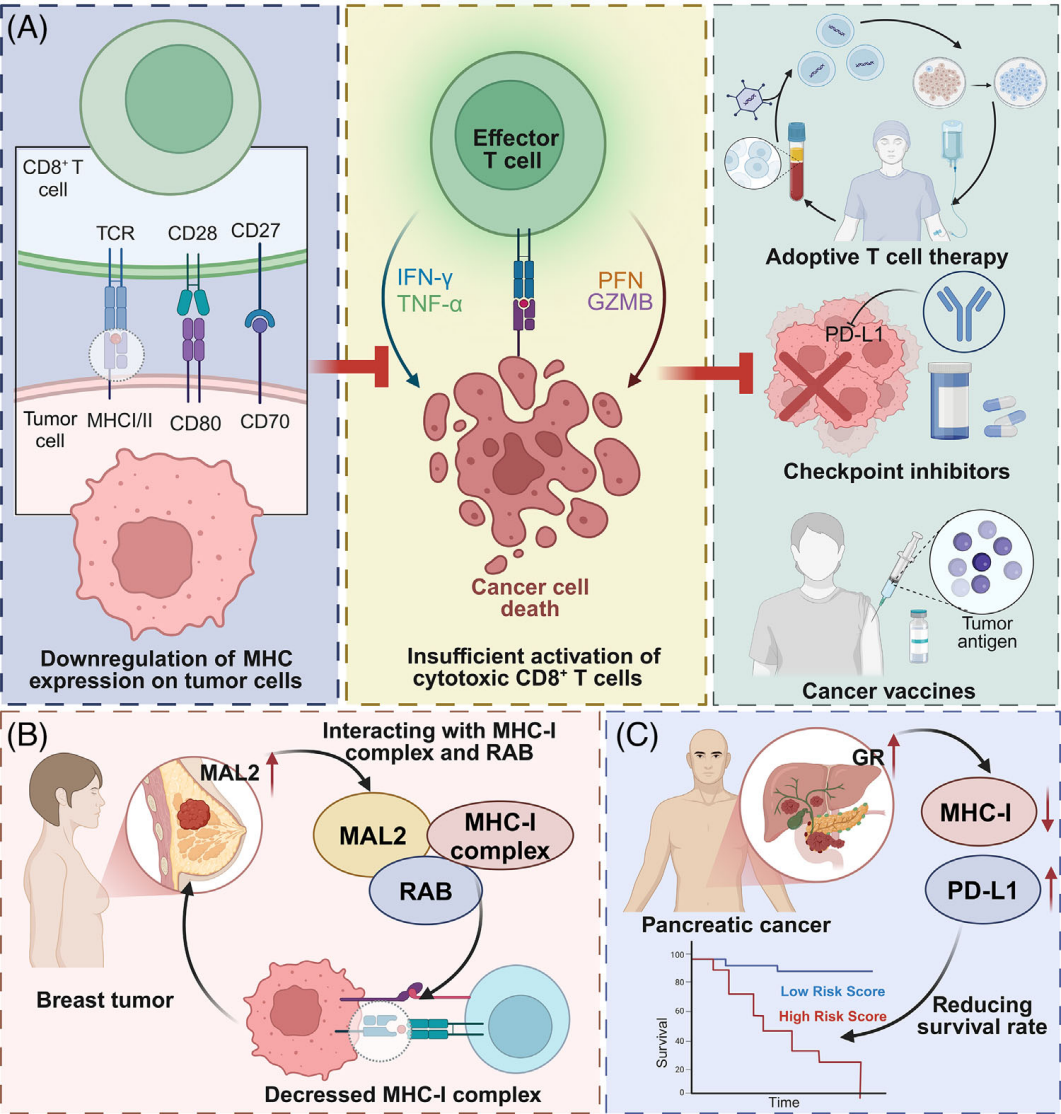
Loss of major histocompatibility complex (MHC) expression in tumours contributes to immune evasion and limits the efficacy of cancer immunotherapy. (A) Tumour cells with reduced surface expression of MHC‐I/II molecules fail to adequately present tumour antigens to CD8⁺ T cells, resulting in insufficient activation of TCR signalling. The absence of co‐stimulatory signals such as CD80 and CD70 further impairs CD8⁺ T cell activation. Consequently, effector T cells produce lower levels of interferon‐gamma (IFN‐γ) and TNF‐α, and release reduced amounts of perforin and granzyme B (GZMB), ultimately weakening their cytotoxic capacity and allowing tumour cells to evade immune destruction. This impaired immune activation compromises the therapeutic outcomes of adoptive T cell transfer, immune checkpoint blockade targeting PD‐L1, and cancer vaccines based on tumour antigens. (B) In breast cancer, the membrane protein MAL2 is upregulated and facilitates the internalization of the MHC‐I complex through its interaction with MHC‐I and the small GTPase RAB. This leads to the removal of MHC‐I from the cell surface and promotes lysosomal degradation, resulting in decreased MHC‐I levels on tumour cells and reduced recognition by CD8⁺ T cells, thereby contributing to immune escape. (C) In pancreatic cancer, GR signalling regulates the expression of both MHC‐I and PD‐L1. Increased GR activity correlates with elevated levels of MHC‐I and PD‐L1, which is associated with reduced overall survival. Kaplan–Meier survival analysis shows that patients with high expression of MHC‐I and PD‐L1 exhibit significantly worse prognosis compared to those with low expression. The images in the figures were created using BioRender (https://www.biorender.com/).

The tumour microenvironment (TME) imposes additional pressures that modulate MHC expression. Immunosuppressive cytokines in the TME, such as TGF‐β, can downregulate MHC molecules and impair antigen presentation. In multiple myeloma, Tregs promote TGF‐β1 production in tumour cells, which suppresses the cGAS–STING pathway and reduces MHC‐I expression while increasing PD‐L1 levels.^38^, ^39^, ^40^ Hypoxia, a hallmark of solid tumours, stabilizes hypoxia‐inducible factors (HIFs) that repress antigen presentation machinery. Chronic immune pressure—such as sustained interferon signalling or upregulation of immune checkpoints—can reinforce MHC downregulation as tumour cells adapt to immune‐mediated selection. Together, these genetic, epigenetic and environmental factors converge to drive MHC loss and facilitate tumour immune escape.^41^ Understanding this complex regulatory network is critical for designing therapies aimed at restoring MHC expression and improving immunotherapy outcomes (see schematic in Figure 1). In contrast to cell‐intrinsic genetic and epigenetic changes, microenvironmental factors such as TGF‐β or hypoxia exert a dynamic and context‐dependent influence on MHC expression. These extrinsic cues enable tumour cells to rapidly adapt to immune pressure, suggesting that modulation of the tumour milieu may offer an indirect yet effective route to reinstate antigen presentation.

## 3 TRANSCRIPTIONAL REGULATION OF MHC EXPRESSION IN SHAPING ANTITUMOUR IMMUNITY

MHC regulation at the transcriptional level is a critical process that governs the expression of MHC, which are essential for immune surveillance and antigen presentation. The transcription of MHC genes is primarily controlled by a set of regulatory elements, including promoter regions, enhancers and transcription factors. Key transcription factors, such as NF‐κB, IRF‐1 and STAT1, play pivotal roles in activating the transcription of MHC‐I and MHC‐II genes in response to signals like IFN‐γ and pro‐inflammatory cytokines. These factors bind to specific DNA motifs within the MHC gene promoters, initiating the transcription of MHC molecules. Additionally, epigenetic modifications, including histone acetylation and DNA methylation, further influence MHC gene expression by altering chromatin accessibility and the recruitment of transcriptional machinery. Transcriptional co‐activators such as NLRC5 for MHC‐I and the class‐II transactivator (CIITA) for MHC‐II also play essential roles by facilitating the recruitment of RNA polymerase and associated factors. Alterations in these regulatory pathways can lead to reduced MHC expression, leading to immune evasion by tumour cells and reduced efficacy of immune responses. Thus, understanding the molecular mechanisms that govern MHC transcription is crucial for developing strategies to modulate MHC expression in cancer immunotherapy.

### 3.1 Key transcription factors

#### 3.1.1 STAT1

STAT1 is a crucial transcription factor activated by IFN‐γ signalling, playing an important role in the regulation of MHC‐I expression. Upon IFN‐γ binding to its receptor, the JAK‐STAT pathway is triggered, leading to the phosphorylation of STAT1 at Tyr701 which promotes its dimerization and nuclear translocation. In the nucleus, STAT1 binds to specific DNA elements within MHC‐I promoters, driving transcription of key components, such as B2M and other antigen processing molecules. Various key proteins have been identified as regulators of STAT1, modulating MHC expression at the transcriptional level and thereby influencing the efficacy of cancer immunotherapy. For instance, the AGC family protein kinases LATS1/2 directly phosphorylate STAT1 at Ser727, promoting nuclear translocation and activation of IRF1/NLRC5, essential for MHC‐I transcription in response to IFN‐γ signalling. Disruption of this pathway reduces tumour susceptibility to CD8^+^ T cell‐mediated cytotoxicity, highlighting LATS1/2 as potential immunotherapy targets in endometrial cancer.^42^ In addition, regulator of G‐protein signalling 1 (RGS1) has been recognized as a critical regulator of cancer immunogenicity. It enhances IFNγ‐STAT1 signalling and MHC‐I expression by promoting ATF3‐mediated activation of the IFNGR1 promoter. This pathway promotes CD8^+^ T cell infiltration and effective antigen presentation, with lower RGS1 expression linked to reduced PD1 inhibitor efficacy and shorter progression‐free survival in Non‐small cell lung cancer (NSCLC) patients.^43^ Likewise, IL11 promotes colorectal cancer (CRC) immune evasion by dampening IFN‐γ‐induced STAT1 phosphorylation via STAT3, thus reducing MHC‐I and CXCL9 expression and CD8^+^ T cell infiltration. Competitive inhibition by IL11 mutein reverses this suppression, enhancing MHC‐I and CXCL9 levels, and could be a targeted strategy for anti‐cytokine therapy.^44^ Interestingly, protein arginine methyltransferase 1 (PRMT1) is identified as a negative regulator of MHC‐I expression, attenuating CD8^+^ T cell‐mediated anti‐tumour responses by suppressing IFN‐γ‐induced STAT1 activation. Targeting PRMT1, either through knockout or pharmacological inhibition with GSK3368715, enhances MHC‐I expression and the effectiveness of anti‐PD‐1 immunotherapy.^45^

#### 3.1.2 NF‐κB

Upon activation by various stimuli, such as pro‐inflammatory cytokines, NF‐κB translocates to the nucleus, where it binds to the promoter regions of MHC genes, enhancing their transcription. Similar to STAT1, several key proteins have been identified that regulate MHC transcription at the transcriptional level, influencing cancer immunotherapy outcomes. Inhibition of Wnt Family Member 7A (WNT7A) disrupts its interaction with Frizzled Class Receptor 5 (FZD5), deactivating the Wnt/β‐catenin signalling pathway and facilitating the nuclear translocation of p65. This activation of the NF‐κB pathway promotes MHC‐I gene transcription, enhancing CD8^+^ T cell infiltration and antitumour immunity. Targeting WNT7A may thus improve the efficacy of T cell‐based immunotherapies.^46^ In addition, TNF receptor associated factor 3 (TRAF3), identified as a suppressor of NF‐κB and a negative regulator of MHC‐I, enhances survival and response to ICB therapy upon knockout. SMAC mimetic birinapant, by mimicking the transcriptional effects of Traf3‐knockout, upregulates MHC‐I and boosts T cell‐mediated cytotoxicity, augmenting ICB efficacy.^47^

#### 3.1.3 IRFs

IRFs play a crucial role in activating MHC expression by directly interacting with the promoter regions of MHC genes, particularly MHC‐I. In response to inflammatory signals, such as IFN‐γ, IRFs facilitate the transcription of MHC molecules, enhancing antigen presentation and immune surveillance. For instance, loss of IRF2 in various human cancers is associated with decreased MHC‐I expression and diminished recognition by CD8⁺ T cells, promoting resistance to checkpoint inhibitor immunotherapy. Gene editing of IRF2 diminishes transcription of MHC‐I pathway genes and surface complex levels. However, this effect is reversible; interferon stimulation can activate IRF1, restoring MHC‐I expression and immunotherapy responsiveness.^48^ Nucleophosmin 1 (NPM1), a nucleolar phosphoprotein, suppresses tumour immunogenicity by binding to IRF1 and preventing its association with the NLRC5 and CIITA promoters. This impairs the transcription of MHC‐I and MHC‐II. Loss of NPM1 enhances CD8^+^ T cell infiltration, activation, and specific T cell killing, indicating its role in promoting immunosuppressive tumour microenvironment (ITME).^49^

**FIGURE 2:**
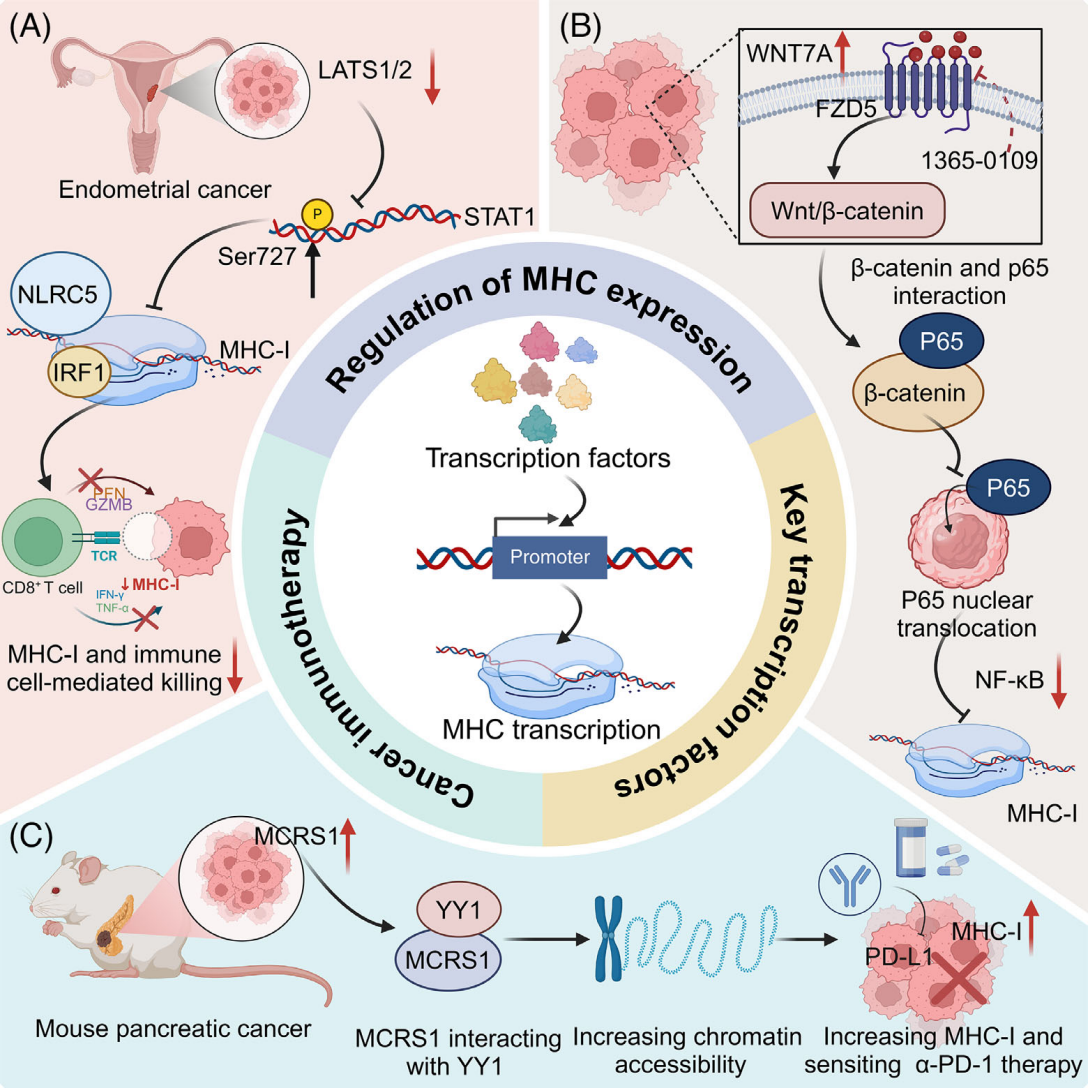
Transcriptional regulation of major histocompatibility complex (MHC)‐I expression in cancer and its impact on immune evasion and immunotherapy. (A) In endometrial cancer, loss of LATS1/2 reduces phosphorylation of STAT1 at Ser727, impairing its nuclear translocation and transcriptional activity. This leads to downregulation of MHC‐I expression. In parallel, impaired activation of IRF1 and NLRC5 further suppresses MHC‐I gene transcription. As a result, tumour cells present fewer antigens to CD8⁺ T cells, reducing the production of cytotoxic mediators such as IFN‐γ and granzyme B (GZMB), and thereby escaping immune‐mediated killing. (B) In various cancers, upregulation of WNT7A and its receptor FZD5 activates Wnt/β‐catenin signalling. β‐catenin binds to the transcription factor p65, preventing its nuclear translocation and inactivating the NF‐κB pathway. Since NF‐κB is a key driver of MHC‐I transcription, this results in decreased MHC‐I expression and promotes immune escape by reducing antigen visibility to T cells. (C) In a mouse model of pancreatic cancer, MCRS1 is upregulated and interacts with YY1, a transcription factor, to enhance chromatin accessibility at MHC‐I gene loci. This facilitates transcriptional activation of MHC‐I and increases tumour cell visibility to cytotoxic T cells. The elevated MHC‐I expression enhances sensitivity to PD‐1 immune checkpoint blockade, suggesting that targeting the MCRS1–YY1 axis may improve immunotherapy efficacy in pancreatic cancer. The images in the figures were created using BioRender (https://www.biorender.com/).

In addition to the above transcription factors, other transcriptional regulators have been identified to regulate MHC expression, further influencing tumour immunogenicity and response to immunotherapy. Microspherule protein 1 (MCRS1) enhances MHC‐I expression and T cell‐mediated immunity in pancreatic cancer by interacting with YY1, a transcription factor, to increase chromatin accessibility at MHC‐I gene loci. This regulation boosts T cell infiltration and responsiveness to α‐PD‐1 therapy, improving survival in pancreatic and lung cancer patients.^50^ (Figure 2).

### 3.2 Epigenetic regulation

Epigenetic regulation plays a crucial role in modulating MHC expression, influencing immune responses in cancer. Modifications such as DNA methylation, histone modification, and chromatin remodelling can either suppress or enhance MHC expression. Epigenetic silencing via polycomb complexes plays a critical role in transcriptional repression through histone modification. The polycomb repressive complex 2 (PRC2) plays a critical role in the immune evasion of cancer cells by transcriptionally silencing the MHC‐I antigen processing pathway through bivalent histone modifications (H3K4me3 and H3K27me3) at gene promoters. This epigenetic mechanism, normally active in embryonic development, is co‐opted by cancers to suppress MHC‐I expression and limit T cell‐mediated immunity, highlighting a potential target for enhancing immunotherapy efficacy.^51^ Similarly, in triple‐negative breast cancer (TNBC), particularly in the mesenchymal subtype, transcriptional suppression of MHC‐I is mediated by H3K27me3 orchestrated by PRC2. Pharmacological intervention of PRC2 components, EZH2 or EED, effectively restores MHC‐I expression and enhances chemotherapeutic outcomes, highlighting the potential of PRC2 inhibitors in enhancing immunogenicity and therapy response in TNBC.^52^ In human melanoma, PRC1 subunit PCGF1 represses MHC‐I expression by promoting ubiquitination at H2AK119 on MHC‐I promoters, while the deubiquitinating enzyme BAP1 enhances MHC‐I expression by removing this mark. Depletion of PCGF1 increases MHC‐I levels across multiple tumour lines, improving T cell‐mediated tumour cell elimination. This delineates a critical epigenetic mechanism by which tumours evade immune detection.^53^

Importantly, WD Repeat Domain 5 (WDR5) is implicated in the dual regulation of tumour growth and immune evasion through its epigenetic control over MHC‐I expression and immunosuppressive pathways in pancreatic cancer. Elevated WDR5 levels correlate with reduced chemotherapy or immunotherapy response but enhance H3K4me3 deposition at MHC‐I promoters, boosting MHC‐I transcription.^54^ Additionally, BET inhibition, particularly targeting Bromodomain containing 4 (BRD4), enhances MHC‐I expression and CD8^+^ T cell‐mediated antitumour immunity in head and neck squamous cell carcinoma (HNSCC). BRD4 inhibits MHC I expression by recruiting G9a, a common histone methyltransferase, which mediates H3K9 methylation. JQ1 treatment inhibits the enrichment of BRD4 and G9a at the promoter of HLA‐A, HLA‐B, and HLA‐C, thereby increasing MHC‐I expression, even under IFN‐γ stimulation.^55^

### 3.3 Non‐coding RNAs (ncRNAs)

**FIGURE 3:**
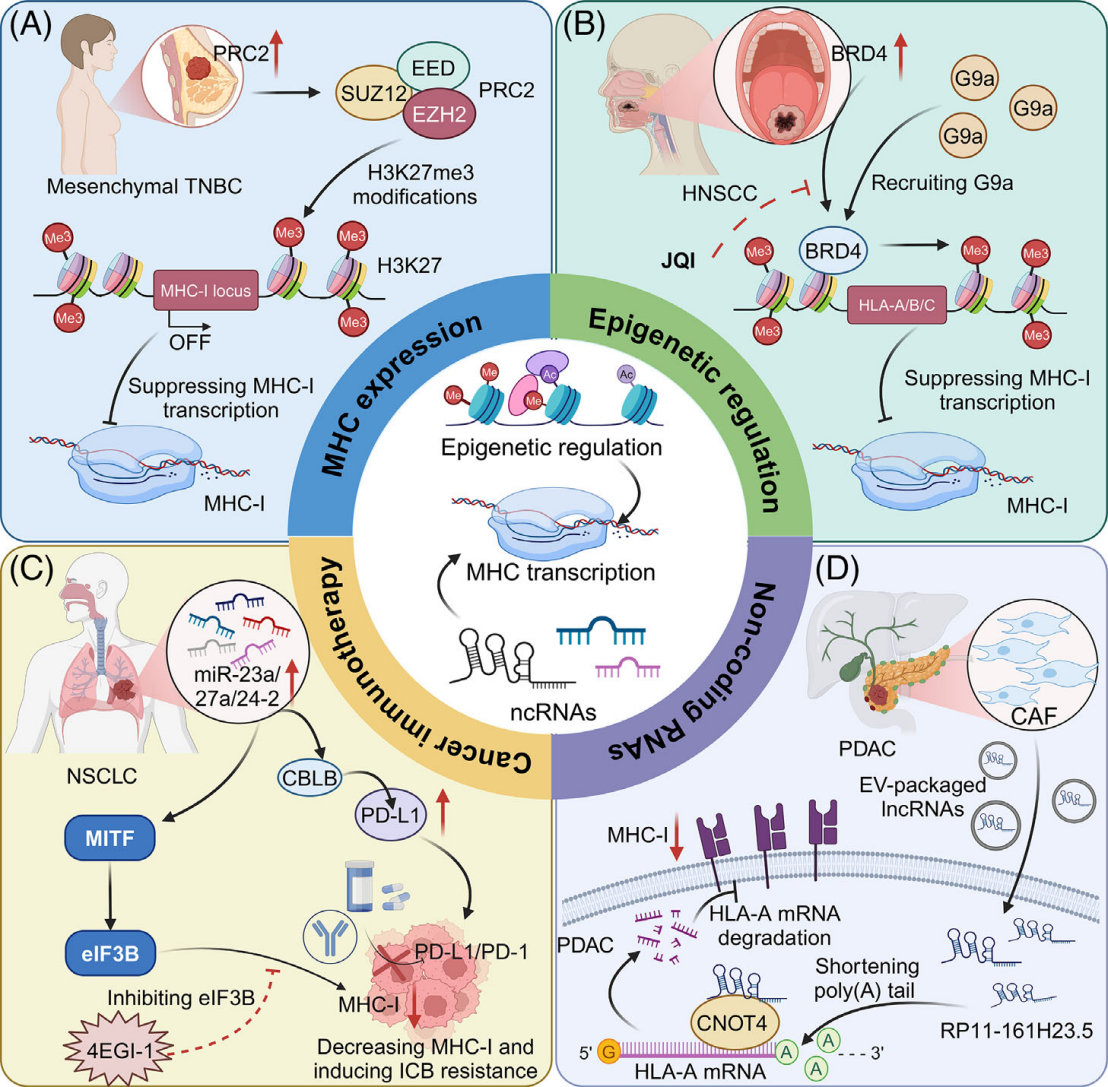
Epigenetic and non‐coding RNA regulation of major histocompatibility complex (MHC)‐I expression and its impact on immune evasion and immunotherapy resistance. (A) In mesenchymal triple‐negative breast cancer (TNBC), the PRC2—composed of SUZ12, EED, and EZH2—induces H3K27me3 at the MHC‐I gene locus. This histone modification leads to chromatin condensation and transcriptional silencing of MHC‐I, thereby impairing tumour antigen presentation to cytotoxic T cells. (B) In HNSCC, BRD4 promotes recruitment of the histone methyltransferase G9a to the HLA‐A/B/C loci, resulting in H3K9 methylation and transcriptional repression of MHC‐I. Pharmacologic inhibition of BRD4 with JQ1 can potentially reverse this suppression and restore MHC‐I expression. (C) In NSCLC, the miR‐23a/27a/24‐2 cluster downregulates CBLB, an E3 ligase that negatively regulates PD‐L1. This leads to PD‐L1 upregulation and reduced MHC‐I expression, contributing to ICB resistance. Meanwhile, the transcription factor MITF inhibits translation initiation factor eIF3B, which also suppresses MHC‐I expression. This suppression can be pharmacologically reversed using 4EGI‐1, a small‐molecule inhibitor of eIF4E–eIF4G interaction, potentially enhancing ICB response. (D) In pancreatic ductal adenocarcinoma (PDAC), CAF release EVs carrying lncRNAs such as RP11‐161H23.5. These lncRNAs promote degradation of HLA‐A mRNA by recruiting the deadenylation factor CNOT4, which shortens the poly(A) tail, leading to transcript destabilization and downregulation of MHC‐I surface expression. This impairs antigen presentation and promotes immune evasion. The images in the figures were created using BioRender (https://www.biorender.com/).

NcRNAs, including microRNAs (miRNAs) and long non‐coding RNAs (lncRNAs), play crucial roles in regulating MHC expression and modulating immune responses in cancer. For instance, in CRC, the chaperone protein calnexin (CANX), essential for MHC‐I assembly, is linked to improved patient survival and is regulated by miR‐148a‐3p. Inhibiting miR‐148a‐3p boosts CANX levels, restoring MHC‐I surface expression and enhancing CD8^+^ T cell‐mediated tumour destruction.^56^ In addition, the miR‐23a/27a/24‐2 cluster contributes to immune evasion in NSCLC through two mechanisms: it upregulates PD‐L1 by targeting CBLB and downregulates MHC‐I by increasing eIF3B, which inhibits MITF. This cluster's expression is sustained by Wnt/β‐catenin signalling. Pharmacological inhibition of eIF3B enhances PD‐1/PD‐L1 blockade sensitivity by boosting MHC‐I levels, offering a new therapeutic approach for NSCLC patients with high miR‐23a/27a/24‐2 expression.^57^ Moreover, the lncRNA inducing MHC‐I and immunogenicity of tumour (LIMIT) enhances tumour immunogenicity by activating the GBP gene cluster, which disrupts the HSF1 association, leading to HSF1‐mediated transcription of MHC‐I machinery. Activation of LIMIT via IFN‐γ or CRISPR enhances MHC‐I expression and immunotherapy efficacy, while its silencing reduces antitumour immunity.^58^ In PDAC, the lncRNA RP11‐161H23.5, packaged within cancer‐associated fibroblast (CAF)‐derived extracellular vesicles, facilitates immune evasion by interacting with CNOT4 to accelerate HLA‐A mRNA degradation, reducing antigen presentation.^59^ LINC00240 is upregulated in cervical cancer, promoting tumour progression by sponging miR‐124‐3p, thereby enhancing STAT3 activation and subsequently inhibiting MHC‐I‐related chain‐A (MICA) expression. This suppression of MICA impairs natural killer T (NKT) cell cytotoxicity, facilitating immune tolerance and cancer advancement.^60^ As summarized in Table 1, miRNAs and lncRNAs modulate gene expression at multiple levels to regulate immune responses (Figure 3).

**TABLE 1:** Transcriptional regulation of major histocompatibility complex (MHC) expression and effects.

Category	Cancer type	Regulator	Mechanism	Effects on MHC	Effects	Clinical implication	Ref.
STAT1 axis	Endometrial cancer	LATS1/2	Promotes STAT1 Ser727 phosphorylation	**↑**	Enhances immune killing	Target for ICB	^42^
	NSCLC RCC	RGS1	Promotes ATF3–IFNGR1–STAT1 axis	**↑**	Increases CD8⁺ T cell infiltration	ICB sensitization	^43^
	CRC	IL11	Inhibits IFNγ–STAT1 signalling	**↓**	Reduces T cell infiltration	Target for cytokine therapy	^44^
	/	PRMT1	Blocks IFNγ‐induced MHC‐I	**↓**	Impairs CD8⁺ T cell killing	PRMT1 inhibitors for ICB	^45^
NF‐κB axis	/	WNT7A	Inhibits NF‐κB via β‐catenin	**↓**	Suppresses CD8⁺ T cell function	Boosts immunotherapy	^46^
	/	TRAF3	Suppressing NF‐κB	**↓**	Alters ICB response	Therapeutic target	^47^
	B‐cell lymphoma	HIV‐1 Tat	Suppresses NF‐κB‐dependent MHC‐II	**↓**	Weakens CD4⁺ T cell response	HIV‐driven lymphoma mechanism	^111^
IRF axis	Melanoma	IRF2 loss	Reducing IRF1	**↓**	Impairs T cell priming	IRF2 as target	^48^
	/	NPM1	Blocks IRF1–Nlrc5/CIITA promoter binding	**↓**	Reduces T cell killing	NPM1 as immunotherapy target	^49^
Other TFs	Pancreas lung	MCRS1	Interacts with YY1, opens chromatin	**↑**	Enhances PD‐1 response	ICB enhancer	^50^
	AML	CtBP, FBXO11	Repress MHC‐II genes, degrade CIITA	**↓**	Enables relapse post‐transplant	tsMHC‐II restoration rationale	^126^
Epigenetic regulators	TNBC	PRC2	H3K27me3	**↓**	Limits T cell immunity	PRC2 inhibition	^52^
	Melanoma	PCGF1	Ubiquitylates H2AK119 at MHC‐I promoters	**↓**	Reduces T cell killing	/	^53^
	Pancreas	WDR5 loss	Inhibits H3K4me3 at MHC‐I promoter	**↓**	Promotes immune evasion	Enhances ICB	^54^
	HNSCC	BRD4	Recruits G9a	**↓**	Lowers CD8⁺ infiltration	BET inhibitor + ICB	^55^
ncRNAs	CRC	miR‐148a‐3p	Targets CANX–MHC‐I	**↓**	Weakens CD8⁺ attack	Immunotherapy target	^56^
	NSCLC	miR‐23a/27a/24‐2 cluster	Targets MITF–eIF3B	**↓**	Causes ICB resistance	Therapeutic target	^57^
	/	LIMIT	Activates GBP–HSF1 axis	**↑**	Enhances MHC‐I expression	Potential target	^58^
	PDAC	RP11‐161H23.5	Promotes HLA‐A mRNA degradation	**↓**	Reduces immune response	Novel intervention	^59^
	Cervical cancer	LINC00240	miR‐124‐3p sponge, induces STAT3	**↓**	Inhibits NKT cell killing	Target in cervical cancer	^60^

## 4 LYSOSOMAL AND POST‐TRANSLATIONAL MODIFICATION‐MEDIATED REGULATION OF MHC DEGRADATION IN SHAPING ANTITUMOUR IMMUNITY

### 4.1 Lysosome‐mediated degradation of MHC

Lysosome‐mediated degradation is essential for regulating the turnover of cellular proteins, including MHC molecules. This process involves the fusion of lysosomes with autophagosomes or endosomes, where internalized or damaged MHC molecules are broken down by acidic hydrolases. The degradation of MHC proteins in this manner can reduce their surface expression, contributing to immune evasion in tumours. In PDAC, immune evasion is closely linked to enhanced autophagy, which promotes the lysosomal degradation of MHC‐I molecules and impairs antigen presentation. A key mediator of this process is the cargo receptor NBR1, which selectively targets MHC‐I for macroautophagy, thereby reducing its surface expression and facilitating resistance to immune checkpoint blockade.^61^ Inhibiting autophagy or lysosomal function can restore MHC‐I levels, enhancing T cell–mediated immune responses.^62^ Furthermore, deletion of receptor‐interacting protein kinase 2 (RIPK2), a key regulator of the tumour microenvironment, disrupts NBR1‐mediated degradation and further augments MHC‐I surface expression, ultimately sensitizing PDAC tumours to anti–PD‐1 immunotherapy.^63^ Notably, NDRG1 upregulates MHC‐I in PDAC cells via lysosomal‐autophagy‐dependent degradation, facilitating CD8^+^ T cell infiltration and activity. This elevation of MHC‐I overcomes resistance to ICB therapies.^64^ Moreover, loss of cholesterol 25‐hydroxylase (CH25H) promotes autophagy, downregulating MHC‐I and reducing CD8^+^ T cell infiltration, accelerating tumour progression. Restoring CH25H expression in PDAC can reduce cell viability under cholesterol‐deficient conditions and slows tumour growth in immune‐competent hosts.^65^ In pancreatic cancer cells, autophagy is linked to Integrin Subunit Beta 4 (ITGB4)‐ BCL2 interacting protein 3 (BNIP3) expression, with ITGB4/BNIP3 activation promoting MHC‐I engulfment by autophagosomes, aiding immune evasion. Subcutaneous tumour graft and orthotopic tumour experiments in mice showed that downregulation of ITGB4 significantly enhanced the therapeutic effect of PD‐1 antibodies on pancreatic cancer.^66^ Moreover, the ER‐resident E3 ligase NFXL1, along with the capture complex composed of NBR1 and the ER‐phagy receptor TEX264, binds to MHC‐I, causing its prolonged retention in the ER and promoting autophagy, a process that correlates with poor patient prognosis. Targeting these complexes may, therefore, enhance the immunogenicity of PDAC and improve therapeutic outcomes.^67^ High tumour‐derived macrophage‐derived progranulin (PGRN) correlates with poor survival, low MHC‐I expression, and immune evasion in PDAC. Inhibition of PGRN restores MHC‐I expression by preventing autophagy‐dependent degradation, slowing tumour growth and enhancing T cell‐mediated cytotoxicity.^68^

In addition to pancreatic cancer, MHC loss affects malignant behaviours and immunotherapy responses in multiple types of cancer. PACSIN1, an oncogene overexpressed in gastric cancer, especially in immunologically cold tumours, suppresses antitumour immunity by promoting lysosomal fusion and selective autophagy of MHC‐I. This reduces antigen presentation and CD8^+^ T cell infiltration. PACSIN1 deficiency enhances antigen presentation, increases CD8^+^ T cell infiltration, and improves response to ICB, thereby inhibiting tumour growth and liver metastasis.^69^ In addition, IRGQ plays a crucial role in the quality control of MHC‐I molecules by directing misfolded MHC‐I to lysosomal degradation via its interactions with GABARAPL2 and LC3B. In the absence of IRGQ, misfolded MHC‐I accumulates and is transported to cell surface, where it triggers immune responses. This enhanced immune recognition leads to increased CD8^+^ T cell reactivity, resulting in improved survival rates in both mice and human patients with hepatocellular carcinoma (HCC).^70^ Importantly, CXCL1 is elevated in CRC and promotes immune evasion through autophagy‐mediated MHC‐I degradation, highlighting CXCL1 as prospective immunotherapy target in CRC.^71^ Moreover, CEMIP, an oncogene, downregulates MHC‐I on CRC cell surfaces by acting as an adaptor that facilitates MHC‐I's clathrin‐dependent endocytosis, leading to its lysosomal degradation. This process impairs antigen presentation and cytotoxic T cell activity, thereby promoting tumour immune evasion. Inhibition of CEMIP, in combination with ICB, enhances the therapeutic efficacy against CRC by preserving MHC‐I surface expression and improving immune surveillance.^72^ Interestingly, tumour‐intrinsic YTHDF1 promotes immune evasion and ICB resistance by enhancing lysosomal proteolysis of MHC‐I and antigens, thereby impairing immune surveillance. YTHDF1 deficiency curtails this pathway, reducing lysosomal gene translation, which preserves MHC‐I and antigen integrity, converting immunologically cold tumours into responsive hot tumours.^73^

**FIGURE 4:**
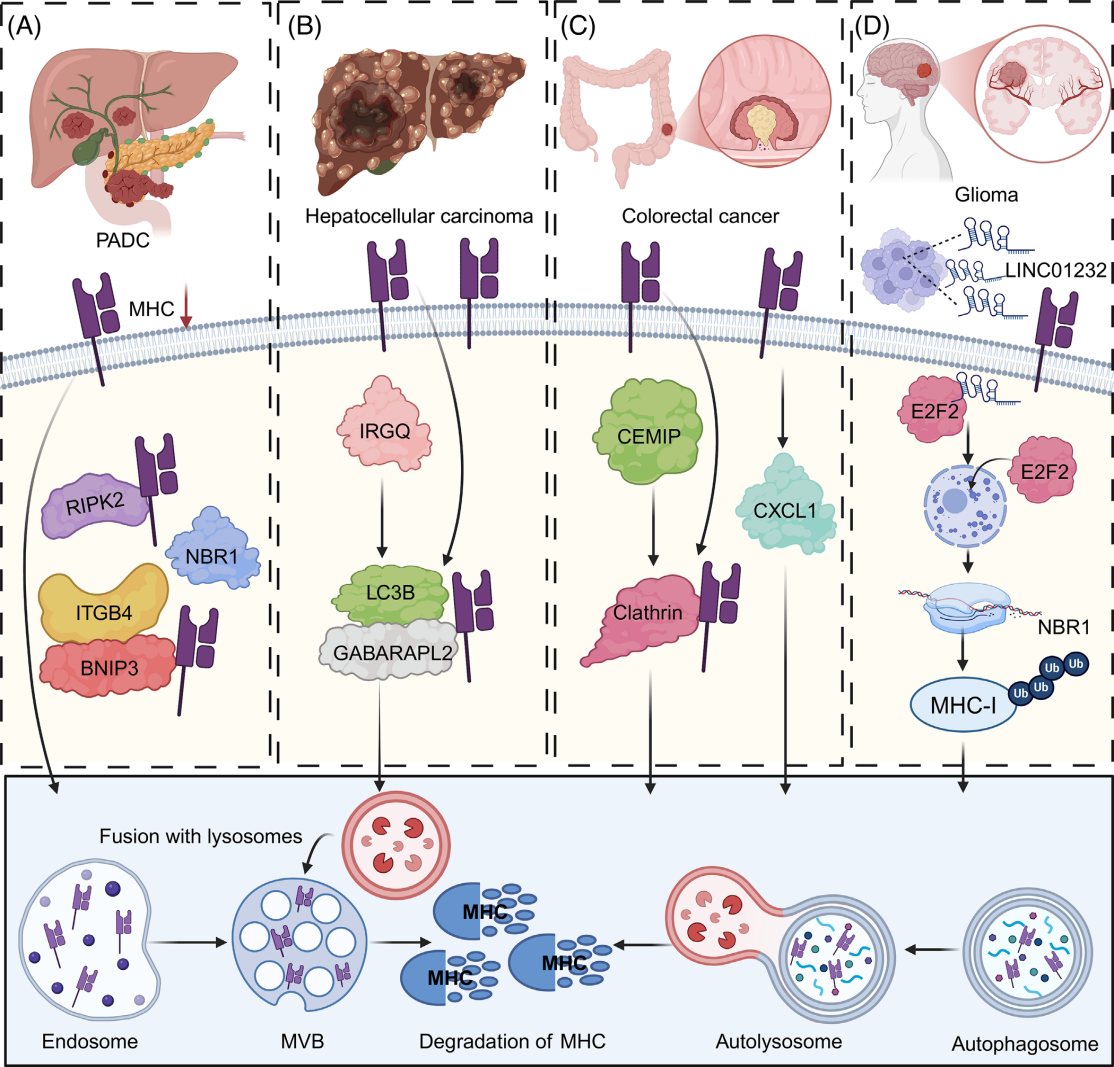
Lysosome‐dependent degradation of major histocompatibility complex (MHC)‐I across cancer types as a mechanism of immune evasion. (A) In pancreatic ductal adenocarcinoma (PDAC), MHC‐I surface levels are reduced due to enhanced internalization and trafficking into the lysosomal degradation pathway. This process is promoted by RIPK2, which interacts with NBR1, integrin β4 and BNIP3. The endocytosed MHC‐I is subsequently targeted to lysosomes, reducing antigen presentation and facilitating tumour immune escape. (B) In hepatocellular carcinoma (HCC), IRGQ activates the autophagy machinery by inducing LC3B and GABARAPL2. These autophagy‐related proteins assist in recruiting MHC‐I into autophagosomes, which later fuse with lysosomes to degrade MHC‐I, thereby impairing CD8⁺ T cell recognition. (C) In colorectal cancer (CRC), CEMIP enhances clathrin‐dependent endocytosis of MHC‐I, directing it toward lysosomal degradation. Additionally, CXCL1 promotes intracellular trafficking that further accelerates MHC‐I loss from the plasma membrane, weakening immune surveillance. (D) In glioma, the long non‐coding RNA LINC01232 is upregulated and increases the expression of the transcription factor E2F2. E2F2 enhances NBR1 expression, which binds to MHC‐I and mediates its ubiquitination and degradation through the autophagy–lysosome system. The lower panel illustrates the common degradation pathway: after internalization, MHC‐I is sorted into early endosomes and MVBs, or directly sequestered into autophagosomes. These vesicles fuse with lysosomes, where MHC‐I is ultimately degraded, leading to impaired antigen presentation and reduced antitumour immunity. The images in the figures were created using BioRender (https://www.biorender.com/).

Interestingly, accumulating evidence suggests that ncRNAs may indirectly influence MHC degradation. In oesophageal cancer, the LINC01592/E2F6/NBR1/MHC‐I axis enhances MHC‐I degradation in autophagolysosomes and reduces its surface expression on cancer cells, facilitating immune evasion by CD8^+^ CTLs and promoting tumour progression.^74^ In addition, HIF1A‐AS2, upregulated by HIF1α through binding to its regulatory region, promotes the autophagic degradation of MHC‐I by enhancing its interaction with the autophagy receptor NBR1, thereby facilitating immune evasion. In HNSCC, elevated HIF1A‐AS2 expression correlates with hypoxic signatures, advanced clinical stages, and reduced CD8^+^ T cell infiltration, revealing a mechanism of hypoxia‐driven immune escape in HNSCC.^75^ In glioma, TAM‐secreted exosomal LINC01232 binds to E2F Transcription Factor 2 (E2F2), promoting its nuclear translocation and enhancing NBR1 transcription. NBR1 then binds to ubiquitinated MHC‐I, facilitating its degradation in autophagolysosomes, enabling tumour cells to evade CD8^+^ T cell attack. Disrupting the LINC01232/E2F2/NBR1/MHC‐I axis inhibits tumour growth and enhances CD8^+^ T cell‐mediated immune response^76^ (Figure 4).

### 4.2 Post‐translational modifications (PTMs)‐mediated degradation of MHC

**FIGURE 5:**
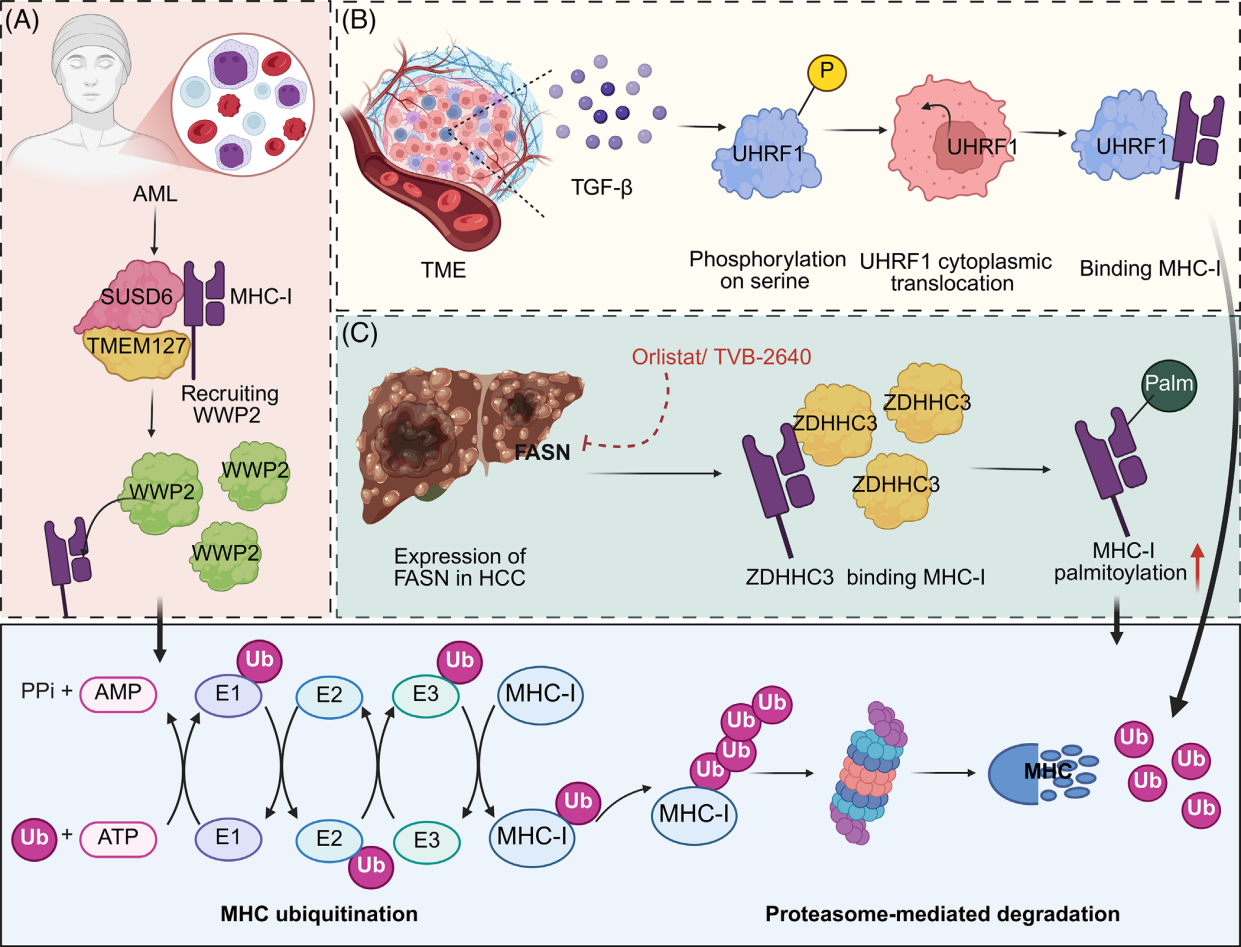
Post‐translational regulation of major histocompatibility complex (MHC)‐I expression and stability in cancer. (A) In acute myeloid leukaemia (AML), MHC‐I on the cell surface is targeted for ubiquitination by a complex composed of transmembrane proteins SUSD6 and TMEM127, which recruit the E3 ligase WWP2. This leads to MHC‐I polyubiquitination and subsequent recognition by the proteasome for degradation. The resulting reduction in MHC‐I impairs tumour antigen presentation and facilitates immune evasion within the TME. (B) In the immunosuppressive tumour microenvironment (TME), TGF‐βsignalling induces phosphorylation of UHRF1 on serine residues, promoting its translocation from the nucleus to the cytoplasm. Once in the cytoplasm, UHRF1 binds to MHC‐I and contributes to its intracellular retention or degradation, thereby suppressing surface MHC‐I expression and impairing T cell‐mediated tumour recognition. (C) In hepatocellular carcinoma (HCC), FASN upregulation enhances palmitoylation of MHC‐I via the palmitoyltransferase ZDHHC3. This lipid modification increases MHC‐I membrane stability. Inhibitors of FASN, such as Orlistat and TVB‐2640, reduce MHC‐I palmitoylation, suggesting a potential strategy to destabilize surface MHC‐I and modulate immune recognition. (D) The bottom panel illustrates the general process of MHC‐I ubiquitination and degradation. MHC‐I is sequentially conjugated with ubiquitin molecules by E1 (activating), E2 (conjugating), and E3 (ligating) enzymes. Polyubiquitinated MHC‐I is then directed to the proteasome, where it is degraded. This pathway provides a general mechanism by which cancer cells reduce surface MHC‐I expression to escape cytotoxic T cell detection. The images in the figures were created using BioRender (https://www.biorender.com/).

PTMs are critical regulatory mechanisms that modulate the functional diversity of proteins, including MHC molecules. Understanding how PTMs regulate MHC turnover is essential for developing targeted cancer immunotherapies, as manipulating these pathways could enhance immune cell maturation, improve T cell responses, and increase the effectiveness of immune checkpoint inhibitors and other immunotherapies. In AML and certain solid tumours, the process of MHC‐I degradation is mediated through a coordinated mechanism involving the interaction of membrane proteins. Specifically, SUSD6 and TMEM127 bind to MHC‐I to form a complex, which then attracts the E3 ubiquitin ligase WWP2. Once recruited, WWP2 ubiquitinates MHC‐I, marking it for degradation in the lysosome. This degradation pathway effectively suppresses antigen presentation, facilitating immune evasion and contributing to the resistance to immune‐checkpoint blockade therapies.^77^ In addition, a major chemokine receptor expressed in many malignant cancer cells, CXCR4, triggers the ubiquitination and subsequent downregulation of MHC‐I heavy chain from the surface, facilitating immune evasion. This process involves direct interaction between CXCR4 and beta2‐microglobulin, critical for MHC‐I heavy chain internalization and targeting to Rab7 in late endosomes.^78^ Moreover, the epigenetic regulator UHRF1 is aberrantly expressed in cancer, where TME signals like TGF‐β induce its phosphorylation and cytoplasmic translocation. This enables UHRF1 to bind to MHC‐I, promoting its ubiquitination and degradation, thereby suppressing the antigen presentation pathway and contributing to cancer immunotherapy resistance.^79^ Interestingly, inhibition of fatty acid synthase (FASN) enhances MHC‐I expression in HCC by reducing palmitoylation‐dependent lysosomal degradation. The enzyme DHHC3, a palmitoyltransferase, directly interacts with MHC‐I, modulating its stability. Pharmacologically targeting FASN, combined with checkpoint inhibitors, boosts MHC‐I levels and CD8^+^ T cell cytotoxicity, showing potential for improved immunotherapy outcomes in HCC^80^ (Table 2, Figure 5) (Table 3).

**TABLE 2:** Lysosomal and ptm‐mediated regulations of major histocompatibility complex (MHC) degradation.

Regulatory type	Cancer type	Regulatory factor	Mechanism	Outcomes	Effects	Clinical significance	Ref.
Lysosome‐mediated degradation	PDAC	NBR1	Employing macroautophagy	MHC‐I degradation	Decreasing T cell‐mediated tumour immunity	NBR1 as a target for PDAC therapy	^62^
	PDAC	RIPK2	Inducing NBR1‐mediated autophagy	MHC‐I degradation	Impeding antigen presentation and cytotoxic T‐cell killing	Basis for combining RIPK2 inhibitor with anti‐PD‐1 therapy	^63^
	PDAC	NDRG1	Inhibiting MHC‐I lysosomal‐autophagy‐dependent degradation	Increasing MHC‐I	Promoting infiltration and activity of CD8 + T cells	NDRG1 as a target to enhance immunotherapy	^64^
	PDAC	CH25H loss	Promoting autophagy	Decreasing MHC‐I	Decreasing CD8+ T cell tumour infiltration	Indicating therapeutic potential	^65^
	Pancreatic cancer	ITGB4	Binding to BNIP3, promoting phagocytosis by autophagosomes	Decreasing MHC‐I	Promoting immune escape	/	^66^
	PDAC	NFXL1, NBR1, TEX264	Binding and prolonging retention time of MHC‐I in ER	MHC‐I autophagic degradation	Decreasing antigen presentation efficiency	NFXL1/TEX264/NBR1 complex as target for immunotherapy	^67^
	PDAC	PGRN	Promoting autophagy‐dependent MHC‐I loss	Decreasing MHC‐I	Decreasing CD8+ T cell infiltration	PGRN as a target for immunotherapy	^68^
	Gastric cancer	PACSIN1	Promoting lysosomal fusion and selective MHC‐I autophagy	Decreasing MHC‐I	Decreasing CD8+ T cell infiltration	Potential of PACSIN1 as a target in GC treatment	^69^
	HCC	IRGQ	Binding to GABARAPL2 and LC3B	Degrading misfolded MHC‐I via lysosomes	Decreasing reactivity of CD8+ T cells	IRGQ as a regulator of MHC‐I quality control	^70^
	CRC	CXCL1	Inducing autophagy‐mediated MHC‐I loss	Decreasing MHC‐I	Promoting immune escape	CXCL1 as a target for immunotherapy	^71^
	CRC	CEMIP	Facilitating clathrin‐mediated MHC‐I internalization	MHC‐I lysosomal degradation	Impairing antigen presentation, limiting CD8⁺ T cell responses	Targeting CEMIP as a strategy for CRC immunotherapy	^72^
	/	YTHDF1	Inducing translation of lysosomal genes	Lysosomal proteolysis of MHC‐I	Impairing tumour immune surveillance	Exosomal CRISPR/Cas9 targeting YTHDF1 enhancing antitumour activity	^73^
	HCC	LAMTOR1	Regulating endocytic pathway through DNM2, enhancing autophagosomes	Decreasing MHC‐II	Reducing antigen expression, decreasing antitumour T cell responses	/	^121^
	/	LINC01592	Cooperating with E2F6, enhancing NBR1 transcription	MHC‐I degradation	Causing cancerous cells to escape from CD8+ CTL immune attack	LINC01592 as a target to enhance CD8⁺ T cell reinfusion efficacy.∖	^74^
	HNSCC	HIF1A‐AS2	Promoting interaction between NBR1 and MHC‐I	MHC‐I autophagic degradation	Reducing infiltration of CD8+ T cells	HIF1A‐AS2 as a potential target for immunotherapy	^75^
	Glioma	LINC01232	Binding E2F2, enhancing NBR1 transcription	MHC‐I degradation	Enabling tumour cells to evade CD8+ T cell attack	LINC01232/E2F2/NBR1/MHC‐I axis as target for therapy	^76^

**TABLE 3:** Ptm‐mediated regulations of major histocompatibility complex (MHC) degradation.

Regulatory type	Cancer type	Regulatory factor	Mechanism	Outcomes	Effects	Clinical significance	Ref.
PTMs‐mediated degradation	Leukaemia and solid cancers	SUSD6/ TMEM127/ WWP2	USD6 forms complex with TMEM127 and MHC‐I, recruiting WWP2 to ubiquitinate MHC‐I	MHC‐I degradation	Suppressing antigen presentation	SUSD6/TMEM127/ WWP2 axis as a potential therapeutic target	^77^
	/	CXCL12	Interacting with B2M, inducing ubiquitination of MHC‐I heavy chain	Decreasing MHC‐I	Increasing tumour evasion	CXCL12 as a potential target for immunotherapy	^78^
	/	UHRF1	Phosphorylation UHRF1 to induce its cytoplasmic translocation	MHC‐I degradation	Suppressing antigen presentation pathway	UHRF1 as a potential target to synergize with immunotherapy	^79^
	HCC	FASN	Increasing MHC‐I palmitoylation	MHC‐I degradation	Decreasing antigen‐specific CD8+ T cell cytotoxicity	Potential of FASN inhibitor–immunotherapy combination	^80^

## 5 STRUCTURAL DEFECTS IN MHC GENES AND THEIR THERAPEUTIC IMPLICATIONS

While many tumours exhibit dynamic and reversible MHC downregulation through transcriptional repression, post‐translational modification, or epigenetic silencing, a subset acquires irreversible genetic alterations that lead to structural loss of MHC molecules. These lesions disrupt antigen presentation at the genomic level and are increasingly recognized as major contributors to both primary and acquired resistance to immunotherapy. Unlike regulatory suppression that may be reversed by inflammatory signals such as interferon‐γ, these structural aberrations result in permanent loss of MHC surface expression and are unresponsive to conventional immunostimulatory therapies.

### 5.1 B2M mutations and LOH

Among the most well‐characterized irreversible mechanisms is inactivation of the β2‐microglobulin (B2M) gene, which encodes the light chain essential for proper folding and surface stability of MHC‐I complexes. Loss‐of‐function mutations, gene deletions, or loss of heterozygosity (LOH) involving B2M impair MHC‐I presentation, rendering tumour cells invisible to CD8⁺ T lymphocytes.^81^ B2M loss has been frequently observed in patients who show initial sensitivity to ICB but subsequently relapse, particularly in melanoma, lung cancer, and colorectal cancer.^82^, ^83^, ^84^ For instance, analysis of tumour biopsies revealed that B2M LOH is significantly more abundant in ICB‐resistant patients and correlates with unfavourable survival outcomes. Complete B2M loss was observed exclusively in non‐responders, impairing antigen presentation and contributing to resistance to anti‐CTLA‐4 and anti‐PD‐1 therapies.^85^

### 5.2 HLA gene alterations

In addition, somatic mutations in MHC‐I heavy chain genes (HLA‐A, ‐B, ‐C) can interfere with peptide binding or disrupt structural integrity, further compromising antigen presentation. In a subset of MSI‐high colorectal cancers, reduced expression of MHC‐I genes (HLA‐A, ‐B, ‐C) is associated with impaired lymphocyte infiltration, compromised antigen presentation and worse prognosis, despite high tumour mutation burden.^86^ Another prominent mechanism involves LOH at chromosome 6p, which encodes the HLA class I region. This HLA‐LOH event often results in haplotype‐specific antigen loss, selectively eliminating alleles that present immunogenic neoantigens while retaining less immunogenic variants.^87^ HLA‐LOH is common in diverse cancers, including NSCLC, lung adenocarcinoma, breast cancer and glioblastoma (GBM), and has been independently associated with poorer survival and diminished immunotherapy responsiveness in large‐scale clinical datasets.^87^, ^88^, ^89^, ^90^

### 5.3 Therapeutic bypass strategies

The clinical implications of these structural aberrations are profound. Tumours with irreversible MHC loss exhibit intrinsic resistance to CD8⁺ T cell‐based immunotherapies, including PD‐1/PD‐L1 and CTLA‐4 blockade and adoptive T cell therapies. Given their resistance to immune‐mediated selection, such tumours are prone to immune escape and often underlie post‐treatment relapse. Therefore, comprehensive genomic profiling of B2M, HLA alleles and antigen‐processing machinery is critical for identifying patients unlikely to benefit from T cell‐centric strategies. In such cases, alternative immune interventions may be warranted. Emerging therapeutic approaches aim to bypass the requirement for MHC‐mediated recognition. These include NK cell‐based therapies, such as cytokine‐induced or CAR‐engineered NK cells, which can detect “missing‐self” phenotypes via the absence of MHC‐I. For example, researchers have reprogrammed T cells into induced T‐to‐natural killer cells (ITNKs) by knocking out BCL11B, endowing them with the combined cytotoxic properties of both T effector and NK cells. These ITNKs, along with CAR‐engineered ITNKs, selectively lyse a broad range of cultured cancer cells and suppress tumour growth in xenograft models.^91^ A phase I clinical study (NCT03882840) evaluating the infusion of ITNK/CAR‐ITNK cells into patients with MHC‐I–low tumours has provided preliminary evidence of their therapeutic efficacy. Additionally, macrophage‐directed agents and innate immune agonists are being investigated to promote antigen‐independent immune clearance. For example, peptidic bispecific antibodies (pBsAbs) have been developed as an innovative platform to enhance macrophage–cancer cell interactions. By combining EGFR‐targeting peptides with an anti‐SIRP‐α antibody, pBsAbs effectively block the CD47–SIRP‐α “don't eat me” signal, promote antibody‐dependent cellular phagocytosis, and enhance macrophage infiltration into tumour spheroids, highlighting their potential to improve antitumour efficacy.^92^ In addition, STING agonists can induce a phenotypic shift of macrophages from an M2‐like pro‐tumour state to an M1‐like anti‐tumour state, overcoming diverse layers of immunosuppression initiated by tumour cells and synergizing with PARP inhibitors to suppress the growth of advanced BRCA‐mutant ovarian tumours. The therapeutic efficacy of this integration depends on host STING and is mediated by a type I interferon response and CD8⁺ T cells, but does not rely on tumour cell‐intrinsic STING.^93^ Strategically combining these innate immune approaches with biomarkers of irreversible MHC loss holds promise for expanding treatment options in otherwise refractory tumours. Together, a comprehensive understanding of both reversible regulatory suppression and irreversible structural defects is essential for informing rational therapeutic design, improving patient stratification, and ultimately overcoming resistance to cancer immunotherapy.

## 6 ANTIGEN PROCESSING AND PEPTIDE LOADING PATHWAYS SHAPING MHC INTEGRITY AND TUMOUR IMMUNE RECOGNITION

In addition to transcriptional and post‐transcriptional regulation of MHC molecules and structural defects in MHC genes, the antigen presentation pathway critically depends on upstream mechanisms of antigen processing and peptide loading.^8^ For MHC‐I, endogenous proteins—such as aberrant or tumour‐associated proteins—are first degraded by the ubiquitin‐proteasome system into peptide fragments. These peptides are then transported into the endoplasmic reticulum (ER) by the transporter involved in antigen processing (TAP1 and TAP2). Within the ER, the peptides undergo trimming by ER aminopeptidases and are subsequently loaded onto MHC‐I with the assistance of peptide‐loading complex components such as tapasin, calreticulin, and ERp57, and the structural component β2‐microglobulin.^10^, ^94^ Proper folding and peptide loading are essential for the stability and surface expression of MHC‐I molecules. Similarly, MHC‐II‐mediated presentation relies on the endocytic uptake and lysosomal degradation of extracellular antigens. Nascent synthesized MHC‐II binds to the invariant chain (Ii) in the ER, which prevents premature peptide binding. Upon trafficking to late endosomal compartments, Ii is degraded, and peptide loading is facilitated by HLA‐DM, while HLA‐DO modulates this process. Subsequently, MHC‐II is trafficked to the cell surface to engage CD4⁺ T cells.^12^, ^95^

Importantly, defects in any of these antigen processing or loading steps can impair MHC surface expression, even when transcriptional levels are intact. Tumours may harbour mutations or downregulation of proteasome subunits, TAP transporters, or chaperone components such as tapasin, leading to defective peptide loading and MHC‐I instability. Likewise, altered endosomal acidification or reduced HLA‐DM expression can interfere with MHC‐II antigen presentation.^96^ These disruptions represent additional mechanisms by which tumours evade immune detection and may contribute to resistance against immunotherapies such as checkpoint blockade. Beyond MHC levels, defective peptide loading due to abnormalities in components can further compromise antigen presentation, contributing to immune evasion and immunotherapy resistance. For example, tapasin, essential for optimal peptide loading onto MHC‐I, shape the repertoire of surface‐presented antigens. In NSCLC, tapasin expression correlates with improved patient survival accompanied by elevated CD8⁺ T cell infiltration. Loss of tapasin disrupts antigen processing, leading to impaired recognition by CTLs specific for tumour‐associated antigens such as survivin and CEP55, ultimately facilitating immune evasion and tumour progression.^97^ Moreover, mutations in interferon regulatory factor 8 (IRF8) in lymphoma impair the expression of CD74 and HLA‐DM—critical intracellular regulators of MHC‐II antigen processing and peptide loading—resulting in defective antigen loading onto MHC‐II complexes, diminished CD4⁺ T cell activation, and enhanced immune evasion.^98^ Therefore, a comprehensive understanding of both MHC expression and upstream antigen processing is essential for the development of effective strategies to restore tumour immunogenicity.

Antigen presentation defects represent a common mechanism of immune evasion across various malignancies. In diffuse large B‐cell lymphoma with secondary central nervous system involvement, impaired antigen presentation and compromised immune surveillance are frequently observed. A cohort study reported varying degrees of genetic alterations affecting key components of the antigen presentation machinery, including deletions in the MHC locus on chromosome 6p and truncating mutations or deletions in the transcriptional regulator CIITA.^99^ In multiple myeloma, defects in antigen presentation are a hallmark of immune escape, partially driven by the activity of Tregs. Within the tumour microenvironment, Tregs secrete TGF‐β1, which inhibits the cyclic GMP‐AMP synthase–stimulator of interferon genes (cGAS–STING) pathway in myeloma cells. This leads to MHC‐I downregulation and PD‐L1 upregulation, impairing CTL recognition and fostering an immunosuppressive microenvironment. Notably, therapeutic strategies targeting TGF‐β1 or activating the cGAS–STING pathway can restore MHC‐I expression, offering a potential approach to counteract immune evasion in hematologic malignancies.^38^ Furthermore, the progression from monoclonal gammopathy of undetermined significance to multiple myeloma has been linked to dysfunction in the antigen processing and presentation machinery, potentially enabling transformed plasma cells to evade immune surveillance during disease evolution.^100^ In paediatric cancers, antigen presentation defects similarly constitute a major barrier to effective antitumour immunity. Recent immunogenetic profiling across large paediatric cohorts has revealed notable alterations in the HLA system. Although somatic mutations in HLA genes and in those involved in antigen processing and presentation are relatively uncommon, significant loss of heterozygosity affecting both HLA class I and class II loci has been reported—particularly in osteosarcoma, glioblastoma, Ewing sarcoma, and low‐grade glioma. Immunohistochemical and transcriptomic analyses have further demonstrated heterogeneous expression of HLA class I molecules and marked reduced transcription of HLA‐B and TAP genes in many advanced paediatric solid tumours. Moreover, HLA‐II molecules are largely absent from tumour cells, with expression restricted to infiltrating immune cells.^101^ These deficiencies in antigen presentation may reduce tumour immunogenicity and limit the efficacy of T cell‐based therapies, underscoring the need to develop immunotherapeutic strategies that restore or bypass impaired MHC function in paediatric oncology.

## 7 THE MHC‐II‐CD4⁺ T CELL AXIS IN TUMOUR IMMUNITY

### 7.1 CD4⁺ T cells as key regulators of antitumour immunity

CD4⁺ T cells serve as key mediators in orchestrating antitumour immune responses beyond their classical function as helper cells. Upon recognition of tumour‐associated antigens presented via MHC‐II, CD4⁺ T cells can differentiate into distinct effector subsets, including Th1 cells that promote cytotoxic activity through IFN‐γ and TNF‐α production, and Tfh cells that support B cell–mediated humoral immunity.^102^, ^103^ For example, in an orthotopic melanoma model, CD4⁺ T cells were shown to stably suppress or eliminate tumours without the aid of CD8⁺ T cells or other lymphocytes. This effect was mediated primarily through TNF‐α and Fas ligand (FasL). IFN‐γ was also essential, acting directly on tumour cells and indirectly by inducing nitric oxide synthase in myeloid cells. These findings highlight the therapeutic potential of harnessing MHC‐II‐restricted cytotoxic CD4⁺ T cell responses in cancer immunotherapy.^104^ In addition, CD4⁺ TILs can shape the quality of the immune reaction through indirect mechanisms. In humanized models, breast cancer CD4⁺ T cells have been shown to inhibit tumour growth by arresting cancer cell cycle progression at the G1/S phase, while simultaneously enhancing antitumour immunity through the activation of pro‐inflammatory signalling pathways. These findings highlight their dual role as both direct cytotoxic effectors and key modulators of the tumour microenvironment.^105^ In addition, in the tumour microenvironment, activated CD4⁺ T cells enhance CD8⁺ T cell priming, facilitate dendritic cell licensing, and contribute to the formation of tertiary lymphoid structures, thereby amplifying immune surveillance and effector function. For instance, histological analysis in colorectal cancer revealed that high intratumoral infiltration of CD45RO⁺ T cells correlate with favourable prognosis, but this effect is contingent upon sufficient stromal infiltration of CD4⁺ T cell subsets, highlighting the supportive involvement of CD4⁺ T cells in orchestrating effective antitumour cytotoxic responses.^106^

### 7.2 The role of MHC‐II in orchestrating antitumour immunity

MHC‐II molecules are critical mediators of immune surveillance, primarily expressed by APCs such as dendritic cells, macrophages, and B cells.^107^ These molecules specialize in presenting exogenously derived peptides, processed via the endosomal–lysosomal pathway, to CD4⁺ helper T cells. Antigens internalized through phagocytosis or endocytosis are degraded in late endosomes or lysosomes, generating peptides subsequently loaded onto MHC‐II within specialized compartments known as MIIC.^108^ This process is facilitated by the invariant chain (Ii) and HLA‐DM, whose activity is further regulated by HLA‐DO. The interplay between HLA‐DM and HLA‐DO critically influences the repertoire of peptides presented on MHC‐II, shaping T‐cell recognition. Upon reaching the cell surface, peptide–MHC‐II complexes engage TCRs on CD4⁺ T cells, initiating their activation, clonal expansion, and differentiation into subsets such as Th1, Th2, or Tfh cells.^96^

Regulation of MHC‐II expression is tightly controlled during immune cell development and activation, playing a critical role in antigen presentation and the initiation of CD4⁺ T cell responses. In immature cells, ubiquitination of the MHC‐II beta‐chain cytoplasmic tail is pivotal for its endocytosis and localization within multivesicular bodies. Upon maturation, this ubiquitination halts, promoting the accumulation of MHC‐II on the plasma membrane, thus enhancing the cell's antigen‐presenting capacity. This selective control of ubiquitination underscores a unique mechanism by which DCs adjust MHC‐II surface expression in response to maturation signals.^109^ Additionally, the E3 ubiquitin ligase March‐I regulates both the surface expression and function of pMHC‐II. While pMHC‐II levels are elevated on the surface of DCs from mice with MHC‐II ubiquitination defects, their ability to stimulate naive CD4^+^ T cells is impaired, and their IL‐12 secretion in response to LPS stimulation is significantly reduced. This highlights that dysregulated MHC‐II turnover impairs DC development and function.^110^ Beyond cell‐intrinsic pathways, MHC‐II expression is also subject to modulation by extrinsic cues such as inflammatory signals and microenvironmental factors. For instance, human immunodeficiency virus I Tat protein disrupts B cell function by downregulating MHC‐II gene expression, notably HLA‐DRB1 and HLA‐DRB5, through diminished NF‐κB pathway activity. This reduction in HLA‐DR surface expression impairs EBV‐specific CD4^+^ T cell responses, facilitating immune evasion and potentially promoting B cell lymphomagenesis in individuals with human immunodeficiency virus (HIV).^111^ Interestingly, aberrant expression of HLA class II on certain tumours can also serve as a hallmark of malignant phenotype and is often connected to negative clinical results. Recent studies have shown that tumour‐intrinsic MHC‐II expression can paradoxically contribute to immune evasion. In breast cancer, MHC‐II⁺ tumour cells in tumour‐draining lymph nodes (TDLNs) were found to lack costimulatory signals, thereby promoting regulatory T cell (Treg) expansion and suppressing CD4⁺ effector responses, ultimately facilitating lymph node metastasis.^112^ Similarly, in the context of anti‐PD‐1 immunotherapy, MHC‐II⁺ tumours displayed initial immune activation with CD4⁺ T cell recruitment but developed adaptive resistance via upregulation of alternative inhibitory receptors such as Lag‐3 and FCRL6.^113^ Together, MHC‐II expression plays a dual role in immune regulation: it is essential for initiating robust CD4⁺ T cell responses, but its dysregulation—through aberrant expression or impaired turnover—can paradoxically promote immune evasion. In tumours, intrinsic MHC‐II may initially activate immunity yet ultimately facilitates immunosuppression by expanding regulatory T cells and driving adaptive resistance. Therefore, precise and context‐dependent regulation of MHC‐II is crucial to balance effective antitumour immunity while preventing immune escape.

In cancer, although MHC‐II expression is typically restricted to APCs, aberrant MHC‐II expression on tumour cells—termed tumour‐specific MHC‐II (tsMHC‐II)—has been documented in various tumour types, including melanoma, breast cancer, PDAC, and classical Hodgkin lymphoma (cHL). For instance, immunohistochemistry, laser‐capture microdissection, and studies of TNBC cell lines have revealed tsMHC‐II expression in TNBC tumour cells, correlating with enhanced infiltration by B and T cells and an improved antitumour immune response, resulting in reduced relapse rates and prolonged progression‐free survival.^114^ Similarly, ectopic MHC‐II expression in PDAC facilitates antitumour immunity by promoting cytotoxic responses from CD4⁺ and CD8⁺ T cells against MHC‐II‐positive tumour cells.^115^ Additionally, in cHL, a malignancy frequently characterized by loss of MHC‐I expression, tsMHC‐II expression enhances CD4⁺ T cell‐mediated antitumour immunity and improves the therapeutic efficacy of anti‐PD‐1 monoclonal antibodies.^116^ The expression of tsMHC‐II is commonly induced via interferon‐gamma (IFN‐γ) signalling through the STAT1–CIITA pathway, enhancing tumour immunogenicity.^117^ Additionally, recent studies have implicated the Hippo signalling pathway as a putative modulator of MHC‐II expression in melanoma.^118^ Analysis of 60 melanoma cell lines has identified a distinct bimodal pattern of MHC‐II expression, revealing subsets capable of constitutive or IFN‐γ‐inducible expression.^119^ Moreover, tsMHC‐II expression is regulated by epigenetic mechanisms. In CRC, IFN‐γ treatment reveals variable tsMHC‐II inducibility, influenced by EZH2‐mediated chromatin remodelling at the CIITA locus and JAK1 mutations. Targeted epigenetic modulation, including EZH2 and histone deacetylase inhibition, enhances tsMHC‐II expression, offering a strategy to overcome immune evasion and improve immunotherapy responses.^120^ In addition, LAMTOR1, also known as p18, is a type of lysosome‐associated membrane protein, modulates MHC‐II expression on tumour cells via the endocytic pathway, influencing CD4^+^ T cell‐mediated recognition and subsequent CD8^+^ T cell antitumour response. Specifically, LAMTOR1 interacts with the GTPase domain of DNM2 to regulate autophagosome formation, thereby decreasing surface MHC‐II levels and impairing antigen presentation.^121^ Functionally, tsMHC‐II enables direct presentation of tumour‐derived antigens to CD4⁺ T cells, bypassing the need for intermediary APCs and thus facilitating local T‐cell activation within the tumour microenvironment.

Conversely, loss or suppression of MHC‐II expression—driven by oncogenic mutations or transcriptional and epigenetic repression of CIITA—undermines antitumour immunity and promotes immune evasion. For example, the BRAFV600E mutation in papillary thyroid carcinoma (PTC) impairs tsMHC‐II expression via the TGF‐β1/SMAD3 signalling axis, facilitating immune escape.^122^ Hypoxic conditions in tumours can suppress forkhead box O1 (FoxO1) activity, impairing its binding to the CIITA promoter and reducing MHC‐II transcription, consequently promoting immune evasion and tumour progression.^123^ In NSCLC, MHC‐II expression is suppressed by histone deacetylases (HDACs) and phosphorylated extracellular signal‐regulated kinase (ERK), limiting CD4⁺ T‐cell activation and intratumoral immune cell recruitment, potentially explaining resistance to immune checkpoint inhibitors in tumours with low MHC‐II expression. Thus, tsMHC‐II expression plays dual roles in both priming and effector phases of antitumour immunity.^124^

Clinically, tsMHC‐II is gaining recognition as both a prognostic biomarker and a predictor of immunotherapy efficacy. Its presence indicates an immune‐inflamed tumour microenvironment, making it a valuable stratification marker for identifying patients likely to benefit from ICB. Recently, dynamic tumour‐specific MHC‐II immuno‐PET imaging in animal models demonstrated that melanoma cells' tsMHC‐II expression, inducible by IFN‐γ, correlates positively with immunotherapy responsiveness.^125^ In acute myeloid leukaemia (AML), the CtBP complex transcriptionally represses MHC‐II genes, while the E3 ubiquitin ligase complex component FBXO11 promotes degradation of CIITA. Overcoming these repressive mechanisms enhances MHC‐II expression in AML cells, thereby stimulating CD4^+^ T cell activation and anti‐tumour responses.^126^ Therapeutically, strategies aiming to restore or enhance MHC‐II expression—such as IFN‐γ administration, CIITA overexpression, or epigenetic reactivation—show promise for augmenting T cell‐mediated tumour recognition and broadening the therapeutic scope beyond MHC‐I‐restricted approaches. Consequently, deeper understanding and targeted manipulation of the MHC‐II–CD4⁺ T‐cell axis represent a critical frontier in cancer immunotherapy research.

Overall, it is important to distinguish the role of MHC‐II in APCs from its tumour‐intrinsic expression. In APCs such as dendritic cells, macrophages, and B cells, MHC‐II is tightly regulated and necessary for displaying exogenous antigens to CD4⁺ T cells, thereby initiating adaptive immune responses and maintaining immune surveillance and tolerance.^127^, ^128^ In contrast, tsMHC‐II refers to ectopic expression of MHC‐II on malignant cells, enabling them to directly present antigens to CD4⁺ T cells within the tumour microenvironment. While this expression can promote antitumour immunity, it may also contribute to immune evasion by expanding regulatory T cells or inducing T cell exhaustion.^129^ Thus, the function of MHC‐II is highly context‐dependent and differs fundamentally between APCs and tumour cells.

## 8 STRATEGIES FOR ENHANCING MHC EXPRESSION

### 8.1 Pharmacological Strategies for Enhancing MHC Expression

**FIGURE 6:**
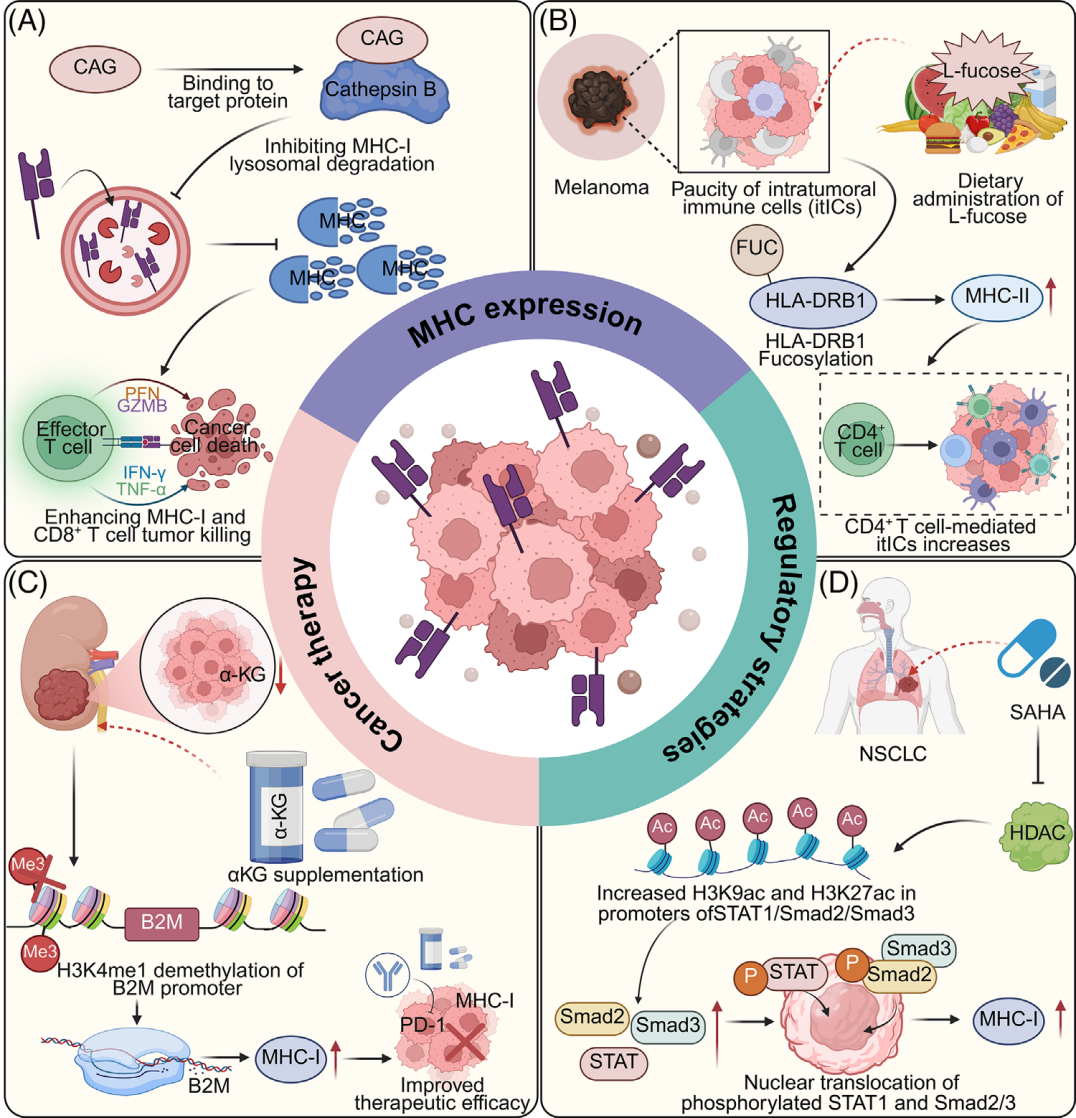
Therapeutic strategies for enhancing MHC expression to improve antitumour immunity. (A) In various tumours, the small molecule CAG binds to a target protein and inhibits the activity of cathepsin B, a lysosomal protease responsible for MHC‐I degradation. This inhibition stabilizes MHC‐I on the tumour cell surface, enhancing antigen presentation and promoting CD8⁺ T cell‐mediated cytotoxicity via perforin and granzyme B (GZMB), ultimately facilitating cancer cell elimination. (B) In melanoma characterized by poor immune infiltration, dietary supplementation with L‐fucose promotes fucosylation of HLA‐DRB1 (a key MHC‐II molecule). This modification increases MHC‐II expression, thereby enhancing CD4⁺ T cell responses and boosting the recruitment of intratumoral immune cells (itICs) into the tumour microenvironment (TME). (C) In kidney cancer, supplementation with α‐ketoglutarate (α‐KG) leads to demethylation of H3K4me1 at the promoter of B2M, a component of MHC‐I. This epigenetic reprogramming increases B2M expression, restores MHC‐I surface levels, and improves antigen presentation, thereby enhancing the efficacy of immune checkpoint inhibitors targeting PD‐1. (D) In NSCLC, treatment with the HDAC inhibitor SAHA increases histone acetylation marks (H3K9ac and H3K27ac) at the promoters of STAT1, Smad2, and Smad3. This enhances the nuclear translocation of their phosphorylated forms, promoting transcriptional activation of MHC‐I‐related genes and enhancing tumour immunogenicity. The images in the figures were created using BioRender (https://www.biorender.com/).

Enhancing the expression of MHC molecules represents a pivotal strategy to augment tumour immunogenicity and bolster immune‐mediated tumour elimination. Recent advances have highlighted approaches to modulate MHC expression through targeted inhibition of degradative pathways, epigenetic regulation, and metabolic reprogramming, which collectively enhance antigen presentation and T cell activation. These strategies underscore the therapeutic potential of MHC modulation in overcoming immune evasion, paving the way for improved efficacy in cancer immunotherapy. For instance, Cycloastragenol (CAG) from *Astragalus membranaceus* binds to cathepsin B, inhibiting its activity, which leads to reduced lysosomal degradation of MHC‐I. This process enhances MHC‐I accumulation on the cell surface, thereby promoting antigen presentation and facilitating the antitumour activity of CD8^+^ T cells. Additionally, combining CAG with PD‐1 antibodies amplifies this effect, boosting immune‐mediated tumour suppression in cancer models.^130^ Moreover, alpha‐ketoglutarate (αKG), a key TCA cycle metabolite, enhances MHC‐I expression in renal cell carcinoma by promoting histone demethylation activities that upregulate B2M transcription via H3K4me1 demethylation in its promoter. This metabolic‐epigenetic modulation boosts antigen presentation and synergizes with PD‐1 blockade, suggesting a strategic combination therapy for overcoming metabolic dysregulation‐induced immune evasion in cancer treatment.^131^ Likewise, low‐dose suberoylanilide hydroxamic acid (SAHA) enhances MHC‐I expression in NSCLC cells by increasing H3K9ac and H3K27ac at the promoters of STAT1, Smad2, and Smad3 through histone deacetylase (HDAC) inhibition. This leads to elevated nuclear translocation of phosphorylated STAT1 and Smad2/3, boosting CD8^+^ T cell activation, proliferation, and cytotoxicity.^132^ Interestingly, the NLRC5‐superactivator (NLRC5‐SA), a smaller fusion protein variant of NLRC5, effectively enhances MHC‐I expression in tumour cells, comparable to full‐length NLRC5. Both constructs expand the repertoire of MHC‐I‐associated peptides, promoting tumour immunogenicity and growth control. This suggests NLRC5‐SA as a viable alternative for boosting antitumour immunity in immunotherapy applications, overcoming the size limitations of using full‐length NLRC5.^133^ Cediranib enhances MHC‐I expression in NSCLC by triggering STAT1 phosphorylation and activating IRF‐1, leading to increased transcription of MHC‐I. This upregulation boosts CD8^+^ T cell infiltration and augments the effectiveness of anti‐PD‐L1 therapy, suggesting cediranib's potential to improve immunotherapy outcomes through modulation of tumour immunogenicity^134^ (Figure 6).

Therapeutic modulation of MHC‐II expression has also emerged as a promising strategy to enhance antitumour immunity across diverse cancer types. In breast cancer, small molecules such as pristane and its derivatives target the malonyl/acetyltransferase domain of fatty acid synthase (FASN), disrupting fatty acid‐mediated MHC‐II silencing and thereby promoting CD4⁺ T cell infiltration.^115^ In melanoma, dietary supplementation with L‐fucose enhances fucosylation of the MHC‐II molecule HLA‐DRB1 on tumour cells, facilitating CD4⁺ T cell recruitment and increasing overall immune cell infiltration. This modification correlates with greater T cell abundance and improved responses to PD‐1 blockade in melanoma patients, illustrating its potential as a biomarker for immunotherapy stratification.^116^ In haematologic malignancies, combination immunotherapies have also shown promise in restoring MHC‐II expression. Flotetuzumab (FLZ), a bispecific DART molecule targeting CD123 and CD3, when used with CAR‐T cells targeting CD123, CD33, or CD371, upregulates MHC‐II through localized IFN‐γ release. This effect has been demonstrated in vitro in THP‐1 acute myeloid leukaemia (AML) cells, in primary AML samples from post‐haematopoietic cell transplantation (HCT) patients with low MHC‐II expression, and in PDX models.^135^ Notably, FLZ was also shown to increase MHC‐II levels in patients with relapsed or refractory AML enrolled in a clinical trial (NCT04582864). Additionally, M234—a LAG‐3 monoclonal antibody that blocks both FGL1 and MHC‐II binding—has been shown to enhance the antitumour activity of CAR‐T cells in hepatocellular carcinoma xenograft models, significantly prolonging survival.^136^ Conversely, in specific tumour contexts, transient inhibition of MHC‐II expression can paradoxically enhance immunity. In melanoma‐bearing mice, blockade of MHC‐II—particularly on dendritic cells—promotes infiltration and activation of CD8⁺ T cells via cDC2‐mediated cross‐priming, and synergizes with immune checkpoint inhibitors to improve therapeutic efficacy.^137^ Collectively, these findings underscore the therapeutic potential of context‐dependent modulation of MHC‐II. Whether through upregulation to restore antigen presentation and enhance CD4⁺ T cell recruitment, or through selective blockade to favour CD8⁺ T cell–driven responses, precise targeting of MHC‐II offers a versatile and powerful approach to optimize immunotherapy across malignancies.

### 8.2 Innate‐Immune Approaches Beyond T Cells

Additionally, while MHC class I downregulation impairs CD8⁺ T cell‐mediated cytotoxicity, it simultaneously renders tumour cells more susceptible to innate immune surveillance, particularly by NK cells. NK cells are endowed with a repertoire of activating and inhibitory receptors that enable them to detect cellular stress and transformation. Notably, the absence or reduction of MHC‐I molecules—a phenomenon known as ‘missing‐self’—releases inhibitory signals mediated through KIRs and NKG2A, thereby promoting NK cell activation and target cell lysis. This compensatory mechanism allows NK cells to surveil and eliminate MHC‐I–deficient tumour cells that escape adaptive immune responses. In TNBC, intratumoral heterogeneity in MHC‐I expression correlates with resistance to anti‐PD‐L1 therapy. However, this heterogeneity also promotes IFN‐γ‐dependent NK cell infiltration. The suppressive receptor NKG2A restrains NK cell activity in MHC‐I‐low regions, and combined blockade of NKG2A and PD‐L1 restores complete responses in mouse models, dependent on both NK and CD8⁺ T cells.^138^ Although innate NK cells can be triggered by MHC‐I loss, their responses are often insufficient. CRISPR‐Cas9 screens under NK and CD8⁺ T cell pressure identified the LUBAC complex—particularly RNF31—as a key regulator of tumour resistance. RNF31 inhibition sensitizes tumours to NK and T cell‐mediated killing through TNF‐driven pathways. Pharmacologic blockade of RNF31 enhances bystander killing of MHC‐I‐deficient tumour cells, offering a potential therapeutic strategy for immune‐refractory cancers.^139^ In addition to NK cells, (TAMs) may compensate for MHC‐I loss when reprogrammed from an immunosuppressive M2‐like phenotype to a pro‐inflammatory M1‐like state. This phenotypic switch enhances their capacity to recognize tumour‐associated damage signals and to mediate direct antitumour effects.^140^ M1‐polarized macrophages can phagocytose tumour cells via Fcγ receptor‐dependent recognition of antibody‐opsonized targets and release pro‐inflammatory cytokines such as TNF‐α and IL‐12, which stimulate local innate immunity and enhance antigen presentation. In tumours exhibiting MHC‐I downregulation, where cytotoxic T cell‐mediated killing is impaired, activated macrophages may facilitate tumour clearance through antibody‐dependent cellular phagocytosis (ADCP) and the recruitment of additional immune effectors, thereby reinforcing local immune activation.^141^, ^142^ In addition to therapeutic targeting, non‐invasive monitoring of tumour‐associated macrophages (TAMs) offers a useful tool for prognosis and treatment planning. A recent study developed mannose‐targeted liposomes carrying Ac4GalNAz, which specifically label TAMs through metabolic glycoengineering and click chemistry. These liposomes showed strong uptake by macrophages in vitro and accumulated in TAMs in a breast cancer model, highlighting their potential for specific, non‐invasive imaging and for guiding macrophage‐targeted therapies.^143^ Thus, targeting macrophage plasticity represents a promising therapeutic strategy to restore antitumour immunity in the context of MHC‐I deficiency.

These insights have spurred the development of non‐T cell‐based therapeutic strategies to target MHC‐I–deficient tumours. NK cell‐based approaches include cytokine‐expanded allogeneic NK cells, CAR‐engineered NK cells, and bispecific NK cell engagers that link tumour‐associated antigens with activating NK receptors such as CD16, thereby enhancing tumour recognition and cytolysis. Furthermore, blockade of inhibitory receptors like NKG2A and KIRs can potentiate NK cell responses in MHC‐I‐low tumours.^144^ On the macrophage axis, innate immune agonists such as STING and TLR agonists are being explored to repolarize TAMs and induce type I interferon responses, which may also support NK cell activation and promote an inflamed tumour microenvironment. Collectively, these approaches highlight the potential of harnessing innate immune mechanisms to overcome immune evasion driven by MHC‐I downregulation. Integrating NK cell‐ or macrophage‐based therapies with immune checkpoint blockade or MHC‐restoring strategies may offer synergistic benefits, particularly in tumours resistant to T cell‐dependent immunotherapy.^145^

## 9 CLINICAL IMPLICATIONS AND TRANSLATIONAL OPPORTUNITIES

Although the mechanistic basis of MHC‐I and MHC‐II downregulation in tumours is increasingly well understood, translating these insights into clinical benefit remains a major challenge. A critical priority is the identification of robust biomarkers that distinguish between reversible and irreversible forms of MHC loss and predict therapeutic responsiveness. Genetic alterations such as B2M mutations or deletions in antigen‐processing machinery (e.g., TAP1/2, ERAP1/2) are associated with clonal and irreversible MHC‐I loss, frequently observed in melanoma, colorectal, and lung cancers, and have been linked to primary resistance to PD‐1/PD‐L1 blockade. In contrast, epigenetic silencing of key transcriptional regulators such as NLRC5 (for MHC‐I) and CIITA (for MHC‐II) appears more common across a broader range of tumours and may be pharmacologically reversible. MHC downregulation often coincides with impaired interferon signalling and broader transcriptional immune escape programs. These immune‐evasive phenotypes can be detected by bulk transcriptomics, but their spatial and subclonal distributions—critical for understanding resistance dynamics—require high‐resolution tools including single‐cell RNA sequencing (scRNA‐seq), spatial transcriptomics and multiplex immunohistochemistry (mIHC).

Numerous clinical trials have begun to validate the feasibility and efficacy of targeting MHC loss through diverse approaches, including small‐molecule inhibitors, epigenetic drugs and cellular engineering. Preclinical studies have shown that inhibition of PCSK9 can restore surface MHC class I levels by preventing lysosomal degradation, thereby enhancing CD8⁺ T cell infiltration and improving responses to PD‐1 blockade.^146^ A phase II clinical trial (NCT05553834) is currently evaluating whether dual therapy with anti‐PCSK9 (alirocumab) and anti–PD‐1 (cemiplimab) antibodies can elicit antitumour responses in patients with metastatic non‐small cell lung cancer who have progressed following first‐line immune checkpoint therapy. Similarly, in a Phase I/II trial (NCT03879798), the combination of valemetostat, a dual EZH1/2 inhibitor, with irinotecan was tested in patients with recurrent small cell lung cancer. This regimen not only showed preliminary clinical efficacy but also increased MHC class I expression during treatment, suggesting a role for EZH1/2 inhibition in reversing immune escape. However, tolerability remains a concern, as several patients experienced dose‐limiting toxicities, underscoring the need for safer combinatorial regimens to harness the immunomodulatory potential of valemetostat.^147^ Notably, another EZH2 inhibitors tazemetostat, currently in phase 1/2 trials (NCT05467748) in combination with PD‐1 blockade, aim to reverse H3K27me3‐mediated repression and restore MHC expression.

In parallel, epigenetic modulators such as decitabine have shown promise in preclinical models by upregulating both MHC class I and II molecules, thereby enhancing leukaemia immunogenicity and sensitizing tumour cells to adoptive T cell therapies.^148^ In a multicentre, single‐arm phase II trial (NCT01690507), elderly patients with acute myeloid leukaemia received decitabine‐based chemotherapy followed by haploidentical lymphocyte infusion. This approach resulted in a complete remission rate of 72.4% and was generally well tolerated, highlighting the potential of epigenetic priming to restore antigen presentation and synergize with T cell‐based therapies. In addition, other epigenetic drugs including DNA methyltransferase inhibitors (e.g., 5‐azacytidine; NCT02260440) and histone deacetylase inhibitors (e.g., entinostat; NCT02437136), can reactivate silenced MHC genes and enhance tumour antigen presentation. In tumours where MHC‐I loss results from enhanced degradation rather than transcriptional repression, inhibitors of autophagy or lysosomal trafficking (e.g., chloroquine, ezurpimtrostat; NCT04214418, NCT05448677) may help stabilize MHC surface expression.^61^, ^149^ Beyond conventional T cell‐based approaches, engineered immune cell therapies are being explored to bypass MHC dependency altogether. In an ongoing phase I trial (NCT03882840), autologous induced T‐to‐natural killer (ITNK) cells—generated via CRISPR/Cas9‐mediated BCL11B knockout—were administered to patients with advanced solid tumours. Early results demonstrated tumour stabilization in six of nine patients, including one partial response, with favourable tolerability. These findings illustrate the feasibility of MHC‐independent strategies for tumours with irreversible antigen presentation loss.^91^ Together, these translational advances reflect a paradigm shift from mechanistic understanding to clinical implementation, aiming to reprogram tumour immunogenicity and reinvigorate antitumour immunity through actionable interventions.

Moreover, mechanisms of MHC loss display distinct prevalence patterns and tumour‐type specificity, with important implications for stratificated therapy. Irreversible alterations such as B2M mutations and HLA class I loss of heterozygosity (LOH) are frequently observed in melanoma, lung cancer, and microsatellite instability–high colorectal cancer, particularly in tumours that relapse after immune checkpoint blockade.^81^, ^82^ These genomic lesions result in permanent MHC‐I loss, limiting the efficacy of CD8⁺ T cell‐based therapies. In contrast, reversible mechanisms—such as epigenetic silencing and lysosomal degradation—are more commonly seen in TNBC, pancreatic cancer, and glioma.^52^, ^63^, ^76^ These are often responsive to pharmacological interventions, including epigenetic modulators or autophagy inhibitors. In addition, certain tumour types, such as leukaemia, hepatocellular carcinoma, and colorectal cancer, exhibit frequent post‐translational modifications—particularly ubiquitination and palmitoylation—that impair MHC expression and facilitate immune escape, thereby presenting additional opportunities for therapeutic intervention.^80^, ^150^ A clear understanding of the dominant mechanism in each tumour type is critical for selecting rational immunotherapy strategies.

In light of these developments, a structured comparison of MHC‐targeted therapeutic strategies is essential to guide their clinical application across diverse tumour contexts. Small‐molecule inhibitors offer pharmacological flexibility, dose controllability and combinatorial potential, but challenges remain, including systemic off‐target effects, potential toxicity, and variable impacts on immune signalling pathways, such as interferon responses and antigen processing.^151^ Approaches like epigenetic modulation or inhibition of lysosomal degradation are particularly suited for tumours with reversible MHC downregulation, as they reactivate endogenous antigen presentation and sensitize tumours to T cell‐mediated killing.^152^, ^153^ By contrast, tumours with irreversible MHC loss—caused by genetic mutations or structural deletions—may require MHC‐independent strategies, such as engineered immune effectors.^32^ Ultimately, stratifying tumours based on the reversibility of MHC loss offers a conceptual and clinical framework to optimize immunotherapy. Restoration‐based strategies should be prioritized in tumours with recoverable MHC expression, while bypass strategies may be better suited for MHC‐deficient tumours refractory to reprogramming, thus maximizing therapeutic efficacy through precision‐guided immune intervention.

## 10 CHALLENGES AND PERSPECTIVES

The regulation of MHC molecules is central to tumour immune surveillance and the efficacy of immunotherapy. However, the intricate mechanisms controlling MHC expression, combined with tumour‐driven immune evasion strategies, pose key hurdles to therapeutic development. These include complexities in MHC regulation, species‐specific differences limiting translational research, compensatory immune escape mechanisms and the safety concerns of restoring MHC expression. Addressing these challenges requires innovative strategies to balance efficacy, specificity, and safety in enhancing tumour immunogenicity.^151^, ^154^

Firstly, the intricate regulatory mechanisms governing MHC expression present a critical challenge in understanding tumour immune evasion and developing effective cancer therapies. Tumour cells often exploit the tight regulation of MHC molecules at transcriptional, post‐transcriptional and post‐translational levels to downregulate MHC‐I expression, thereby escaping CD8^+^ T cell‐mediated immune surveillance. Genetic variations in MHC loci and the influence of tumour microenvironmental factors further contribute to the differential expression of MHC‐I and MHC‐II, complicating efforts to restore antigen presentation.^29^, ^107^ Deciphering these regulatory networks in the context of tumours is essential for unveiling how malignancies evade immune recognition and resist immunotherapy, providing a foundation for targeted interventions to enhance immune responses. Addressing the complexities of MHC regulation requires a combination of advanced multi‐omics profiling and targeted therapeutic development. Multi‐omics approaches, including scRNA sequencing, ATAC‐seq, and proteomics, can provide a comprehensive view of transcriptional and epigenetic mechanisms governing MHC expression in various contexts.^52^, ^118^, ^155^ Additionally, AI‐driven computational models can further accelerate the identification of modulators that enhance antigen presentation, providing a pathway for improved immunotherapy and disease management.^156^

Secondly, the high polymorphism and species specificity of MHC molecules present significant challenges in the development and clinical translation of MHC‐targeted therapies, particularly in the context of cancer.^10^, ^157^, ^158^, ^159^ While preclinical studies have shown encouraging outcomes in counteracting MHC loss and restoring antigen presentation, the substantial differences between MHC molecules across species, such as between mice and humans, limit the direct applicability of findings from animal models to the human immune system.^134^, ^160^, ^161^ Furthermore, variations in MHC expression across genetic backgrounds and disease states introduce additional layers of complexity and uncertainty in the design and interpretation of clinical trials. To address the challenges posed by MHC polymorphism and species specificity in cancer immunotherapy, humanized models, such as mice engineered to express human HLA alleles and immune components, can better recapitulate human‐specific MHC interactions and immune responses, enabling more accurate evaluation of MHC‐targeted therapies.^162^ Additionally, patient‐derived organoid platforms co‐cultured with autologous immune cells provide a human‐relevant in vitro system to study MHC dynamics and tumour‐immune interactions, offering complementary insights to in vivo models and facilitating translational research.^120^, ^163^

Thirdly, although restoring MHC expression enhances tumour immunosurveillance, tumours often exploit alternative immune evasion mechanisms, such as upregulating PD‐L1, to suppress T cell‐mediated antitumour activity. This compensatory escape underscores the limitations of a single MHC restoration strategy in achieving durable tumour control. Tumours can dynamically adapt to therapeutic pressures by modulating immune checkpoints, secreting immunosuppressive cytokines, recruiting regulatory immune cells, or altering antigen processing pathways, thereby creating an immunosuppressive microenvironment that counteracts the benefits of enhanced MHC expression. To address tumour immune escape mechanisms beyond MHC downregulation, combining MHC restoration with ICB, such as anti‐PD‐1/PD‐L1, offers a promising strategy to simultaneously enhance antigen presentation and alleviate T cell inhibition, thereby promoting robust antitumour immunity.^130^, ^164^ Additionally, incorporating co‐stimulatory receptor agonists, such as OX40 or 4‐1BB, can further enhance T cell activation and proliferation, effectively counteracting tumour‐induced immune suppression and achieving sustained immune responses.^165^, ^166^

Fourthly, restoring MHC expression to enhance tumour immune responses holds significant therapeutic potential but presents substantial safety challenges, particularly in controlling immune‐related adverse effects. The upregulation of MHC may inadvertently cause the immune system to misidentify normal cells as foreign, posing a serious risk of autoimmune responses and consequent tissue damage, which could undermine the safety of MHC‐targeted therapies. This phenomenon resembles the dysregulated immune activity observed in autoimmune diseases.^167^ Additionally, excessive immune activation could induce a cytokine storm, resulting in widespread inflammation, organ damage, and, in severe cases, life‐threatening complications. The long‐term effects of MHC restoration remain unclear, as prolonged immune system activation may contribute to chronic inflammation or increase the risk of autoimmune disorders. Epigenetic modulators such as HDAC inhibitors and metabolic reprogramming agents often lack target specificity, potentially inducing widespread transcriptional changes that affect both tumour and normal cells.^168^, ^169^ These off‐target effects may lead to unanticipated toxicities or even promote pro‐tumorigenic pathways in certain contexts. Balancing therapeutic efficacy with these potential risks is a critical challenge in developing safe and effective immunotherapy strategies. To mitigate off‐target immune effects, therapeutic approaches should aim to restore MHC expression specifically in tumour cells while minimizing impact on normal tissues. This could be achieved by employing tumour‐specific delivery systems, such as nanoparticle‐based delivery of gene‐editing tools or small molecules that selectively target tumour cells.^170^, ^171^, ^172^, ^173^ Alternatively, transcriptional activators or epigenetic modulators could be designed to selectively act on the tumour microenvironment, leveraging unique tumour‐specific features, such as hypoxia or abnormal metabolic pathways, to ensure precision targeting. Importantly, comprehensive preclinical and clinical studies are essential to assess the long‐term safety of MHC restoration strategies. Developing predictive biomarkers for immune‐related adverse events could enable early detection and intervention to mitigate risks. Additionally, integrating advanced computational models to simulate immune dynamics could provide insights into potential adverse effects and inform the design of safer therapeutic regimens.^47^

Finally, although bioinformatic analyses based on The Cancer Genome Atlas (TCGA) have greatly advanced our understanding of MHC expression and immune evasion across cancer types due to its large sample size and integrated clinical‐genomic data, limitations such as lack of cellular resolution, tumour heterogeneity, and batch effects can still confound interpretation.^174^ To overcome these limitations, integrating TCGA with single‐cell and spatial transcriptomic technologies, along with applying deconvolution and normalization methods, can improve data interpretation.^175^ Beyond these technical constraints, the inherent biological complexity of tumour heterogeneity poses a major obstacle to effective immunotherapy, particularly regarding MHC expression and antigen presentation.^176^ While bulk analyses often suggest uniform MHC downregulation across tumour populations, emerging evidence indicates that MHC loss can occur in a clonal or subclonal manner, reflecting distinct evolutionary pressures within the tumour ecosystem. Clonal MHC loss, resulting from early genetic or epigenetic events, leads to uniform antigen presentation defects across the majority of tumour cells, often correlating with widespread resistance to T cell–mediated immunity. In contrast, subclonal MHC loss may arise later during tumour progression or in response to immune pressure, generating intratumoral diversity in MHC expression and allowing for localized immune evasion while preserving overall tumour viability. This spatial and cellular heterogeneity in MHC expression has critical implications for immune recognition and therapeutic responsiveness.^138^ Moreover, MHC heterogeneity may contribute to mixed or transient responses to immune checkpoint blockade, complicating therapeutic stratification.

To systematically interrogate the complexity of MHC heterogeneity, advanced technologies including scRNA‐seq and spatial transcriptomics offer powerful platforms for dissecting the transcriptional landscape of MHC expression at high resolution. These approaches can reveal co‐occurring immune evasion programs, identify immune‐excluded niches, and map spatial gradients of antigen presentation across tumour regions. ScRNA‐seq has emerged as a transformative tool in cancer research, offering unprecedented resolution to dissect tumour heterogeneity and uncover rare but clinically relevant subpopulations such as drug‐tolerant persister (DTP) cells and exhausted T cells.^177^, ^178^ By capturing the transcriptomic profiles of individual cells, scRNA‐seq enables precise characterization of malignant and immune cell states, facilitates the identification of prognostic markers such as AIMP1 and CNIH4 in HNSCC, and reveals novel functional roles of key regulators such as the VGSC β3 subunit in glioma.^179^, ^180^, ^181^ In the context of MHC expression in cancer, scRNA‐seq offers a unique advantage in characterizing MHC heterogeneity by quantifying MHC gene expression at the single‐cell level, thereby distinguishing MHC‐deficient tumour subclones and dissecting the diverse MHC expression patterns among immune cells within the tumour microenvironment—an essential step toward understanding immune evasion and optimizing personalized immunotherapy.^182^ In particular, integration of scRNA‐seq with TCR profiling can elucidate how MHC heterogeneity shapes T cell clonality, exhaustion, and functional states.^183^ Single‐cell RNA sequencing (scRNA‐seq) offers powerful cellular‐resolution insights into tumour heterogeneity, enabling the identification of rare but clinically relevant subpopulations such as MHC‐deficient tumour cells or exhausted T cells. Unlike bulk RNA‐seq, which provides robust average expression profiles but obscures intra‐tumoral diversity, scRNA‐seq can resolve the complex transcriptional landscape of individual cells. This makes it particularly valuable for dissecting MHC heterogeneity within the tumour microenvironment. By profiling MHC gene expression at the single‐cell level, researchers can uncover immune‐evasive subsets and their regulatory circuits.^184^, ^185^ However, due to technical noise and dropout effects—especially for low‐abundance genes like MHC class I—rigorous quality control, expression imputation, and integration with spatial or protein‐level data are essential to ensure reliable interpretation.

Furthermore, imaging mass cytometry and multiplex immunohistochemistry allow for spatial validation of MHC expression at the protein level and can resolve the interplay between tumour cells and the immune microenvironment within defined anatomical contexts.^68^, ^186^ Spatial transcriptomics allows for the mapping of MHC‐related gene expression in distinct tumour niches, revealing spatially confined immune evasion strategies.^182^ In addition, genome‐wide CRISPR screens enable unbiased interrogation of gene function by systematically disrupting individual genes and assessing their impact on drug response. This approach can uncover regulators of MHC stability, trafficking, immune evasion, and therapeutic resistance, offering mechanistic insights and revealing actionable targets. When integrated with transcriptomic and clinical data, CRISPR‐based functional genomics can inform the development of combination therapies and precision immunotherapy strategies.^187^, ^188^ Notably, the integration of scRNA‐seq, bulk RNA‐seq, and spatial transcriptomics enables comprehensive characterization of MHC‐driven immune landscapes across cancers. The resulting MHC transcriptional signature (MHC.sig) serves as a pan‐cancer predictor of immunotherapy response and a tool for identifying synergistic therapeutic targets, emphasizing the utility of multi‐omics approaches in MHC‐related research.^189^ Incorporating these tools into studies of MHC regulation will not only improve our understanding of immune escape dynamics but also aid in the development of predictive biomarkers and tailored interventions that account for intratumoral diversity. A comprehensive framework that considers clonal architecture and spatial distribution of MHC expression represents a promising avenue for refining MHC‐targeted precision immunotherapy.^190^

## 11 CONCLUSIONS

Loss or downregulation of MHC molecules is a key mechanism by which tumours evade immune surveillance and develop resistance to immunotherapy. This review synthesizes recent advances in our understanding of the regulatory networks governing MHC expression, with particular emphasis on post‐translational control, intracellular trafficking, and tumour‐intrinsic signalling pathways—areas often underrepresented in prior literature. We also highlight the growing repertoire of therapeutic strategies aimed at restoring antigen presentation, including metabolic reprogramming, epigenetic modulation, inhibition of protein degradation, and gene activation. These approaches offer new avenues to enhance tumour immunogenicity and improve the efficacy of immune checkpoint blockade.

To accelerate clinical translation, future efforts should prioritize the development of robust biomarkers to stratify patients based on the extent and reversibility of MHC deficiency. Such tools will be essential for guiding the rational use of MHC‐modulating agents and their integration with existing immunotherapies. In parallel, systematic evaluation of combinatorial regimens—particularly those involving epigenetic drugs, metabolic modulators, and checkpoint inhibitors—is needed to identify synergistic interactions that maximize efficacy while minimizing toxicity. Additionally, incorporating MHC expression profiling into personalized treatment strategies may enable more precise immunotherapeutic interventions tailored to individual tumour immune phenotypes. Together, these directions will be critical for advancing MHC‐targeted therapies from mechanistic insight to clinical impact.

## AUTHOR CONTRIBUTIONS

Li Cu and Xinyuan Zhao conceptualized the idea of the review. Li Cu, Pei Lin and Yunfan Lin prepared initial drafts of the manuscript. Li Cu, Xinyuan Zhao, Pei Lin, Yunfan Lin and Xu Chen contributed to the writing, graph creation and manuscript improvement. All authors reviewed the manuscript and approved to the final version of this manuscript.

## CONFLICT OF INTEREST STATEMENT

The authors declare no conflicts of interest.

## ETHICAL APPROVAL

Not applicable.
